# Bi_2_Ti_2_O_7_ Quantum Dots for Efficient Photocatalytic Fixation of Nitrogen to Ammonia: Impacts of Shallow Energy Levels

**DOI:** 10.1002/advs.202408829

**Published:** 2024-09-05

**Authors:** Pengkun Li, Runjie Wu, Peishen Li, Shuai Gao, Zeping Qin, Xingjian Song, Wenming Sun, Zhaorui Hua, Qiang Wang, Shaowei Chen

**Affiliations:** ^1^ Laboratory for Micro‐sized Functional Materials & College of Elementary Education and Department of Chemistry Capital Normal University Beijing 100048 China; ^2^ College of Environmental Sciences and Engineering Key Laboratory of Water and Sediment Sciences (MOE) Peking University Beijing 100871 China; ^3^ Department of Chemistry and Biochemistry University of California 1156 High Street Santa Cruz CA 95064 USA

**Keywords:** Bi_2_Ti_2_O_7_ quantum dot, nanosheet, oxygen vacancy, photocatalytic fixation of nitrogen, shallow energy level

## Abstract

Photocatalytic fixation of nitrogen to ammonia represents an attractive alternative to the Haber–Bosch process under ambient conditions, and the performance can be enhanced by defect engineering of the photocatalysts, in particular, formation of shallow energy levels due to oxygen vacancies that can significantly facilitate the adsorption and activation of nitrogen. This calls for deliberate size engineering of the photocatalysts. In the present study, pyrochlore Bi_2_Ti_2_O_7_ quantum dots and (bulk‐like) nanosheets are prepared hydrothermally by using bismuth nitrate and titanium sulfate as the precursors. Despite a similar oxygen vacancy concentration, the quantum dots exhibit a drastically enhanced photocatalytic performance toward nitrogen fixation, at a rate of 332.03 µmol g^−1^ h^−1^, which is 77 times higher than that of the nanosheet counterpart. Spectroscopic and computational studies based on density functional theory calculations show that the shallow levels arising from oxygen vacancies in the Bi_2_Ti_2_O_7_ quantum dots, in conjunction with the moderately constrained quantum confinement effect, facilitate the chemical adsorption and activation of nitrogen.

## Graphical abstract

Bi_2_Ti_2_O_7_ quantum dots exhibit markedly enhanced photocatalytic performance toward nitrogen fixation to ammonia under simulated sunlight, as compared to the bulk‐like nanosheet counterpart, despite a similar oxygen vacancy concentration. This is ascribed to the moderately constrained quantum confinement effect and formation of shallow energy levels in the former.

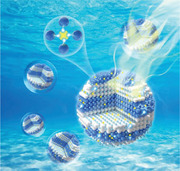

## 1 Introduction

Ammonia is a crucial commodity chemical used extensively in agriculture, chemical production, and energy storage.^[^ ^1^, ^2^, ^3^ ^]^ However, industrial ammonia production relies primarily on the Haber–Bosch process,^[^ ^4^ ^]^ which involves thermal catalytic conversion of hydrogen and nitrogen under extreme conditions (673–873 K, 15–25 MPa). This is an energy‐intensive procedure, consuming ≈2% of the global energy supply annually.^[^ ^5^ ^]^ In addition, this synthesis produces a substantial amount of carbon dioxide that is released to the environment, exacerbating the greenhouse effect. Within this context, there is an urgent need in the development of environmentally friendly and energy‐efficient technologies for ammonia synthesis.^[^ ^6^, ^7^ ^]^

Photocatalytic nitrogen fixation is one of the ideal methods to replace the Haber–Bosch process. Due to the high energy required for the cleavage of the N≡N triple bond (≈941 kJ mol^−1^), the adsorption and activation of inert nitrogen molecules are typically argued to be the key rate‐determining steps of photocatalytic nitrogen fixation under ambient conditions.^[^ ^8^, ^9^ ^]^ A number of strategies have been explored to address this challenge, among which defect engineering has emerged as an effective solution to enhance the efficiency of the photocatalysts, and construction of oxygen vacancies represents a commonly used approach.^[^ ^10^ ^]^ Oxygen vacancies not only enhance light absorption and accelerate charge carrier separation but also provide active sites for N_2_ adsorption and activation.^[^ ^11^, ^12^ ^]^ Additionally, oxygen vacancies can endow photocatalysts with an electron‐rich state and high surface energy. The resulting electron transfer from the e− → *π* ^*^‐orbital (N) to the lowest unoccupied molecular orbital (LUMO) facilitates the enrichment of electron density of N_2_, and hence the adsorption and activation of the molecule.^[^ ^13^ ^]^ Thus far, a variety of methods have been developed for the generation of oxygen vacancies, such as chemical reduction,^[^ ^14^ ^]^ doping,^[^ ^15^ ^]^ etching,^[^ ^16^ ^]^ and size confinement.^[^ ^17^, ^18^, ^19^ ^]^ Of these, size confinement involves diminishing the material size, thereby increasing the surface energy of the material and consequently leading to enrichment of oxygen vacancies. In fact, due to the unique quantum confinement effects, nanosized quantum dot materials have been known to exhibit visible light absorption, abundant surface active sites, and multiexciton effects,^[^ ^20^, ^21^ ^]^ leading to diverse applications, such as photocatalytic water splitting,^[^ ^21^ ^]^ CO_2_ reduction,^[^ ^22^ ^]^ and degradation of organic pollutants,^[^ ^23^, ^24^, ^25^, ^26^ ^]^ among others. Currently, these quantum dots are primarily prepared by millifluidic synthesis^[^ ^27^, ^28^ ^]^ and hydrothermal procedures.^[^ ^12^ ^]^

In these functional nanomaterials, oxygen vacancies can impact the photocatalytic properties by introducing additional energy levels, which can be categorized into shallow and deep energy levels according to the locations.^[^ ^29^ ^]^ Shallow energy levels, which are usually located near the valence or conduction bands, have a large impact on the bandgap of the material and directly contribute to the excitation and conduction of carriers, thus significantly affecting the conductivity and optical properties of the semiconductors. In contrast, deep energy levels are located in the center of the bandgap, which have less influence on carrier transport and electrical properties of the materials.^[^ ^30^ ^]^ Therefore, introduction of oxygen vacancies into shallow energy levels is more favorable for photocatalytic nitrogen fixation. These shallow energy levels can enhance the photo absorption of the materials and promote electron‐hole pair separation, thus improving the photocatalytic activity. The depth of the energy levels are generally identified by using photophysics techniques, such as diffuse reflectance spectroscopy (DRS), photoluminescence (PL) spectroscopy, and photocurrent response (i–t) measurements.^[^ ^31^ ^]^ Notably, it remains a challenge to produce shallow energy levels, and size engineering represents a unique, viable strategy.

Pyrochlore materials have emerged as effective photocatalysts for a wide range of applications, where the size confinement effect has been commonly employed to engineer oxygen vacancies.^[^ ^32^ ^]^ In this study, we used titanium sulfate as the titanium source and bismuth nitrate as the bismuth source and employed a one‐step hydrothermal method to prepare pyrochlore Bi_2_Ti_2_O_7_ quantum dots (BTO‐Q) and bulk‐like Bi_2_Ti_2_O_7_ sheets (BTO‐S) with a similar oxygen vacancy concentration. Bi_2_Ti_2_O_7_ is composed of BiO_4_ tetrahedra and TiO_6_ octahedra, in the space group of Fd3m.^[^ ^33^ ^]^ Results from ab initio molecular dynamics simulation showed enhanced stability of oxygen vacancy‐rich BTO as compared to pristine BTO. Notably, without any sacrificial agent, BTO‐Q exhibited an ammonia production rate of 332.04 µmol g^−1^ h^−1^ in a nitrogen atmosphere under simulated sunlight, which was over 77 times higher than that of BTO‐S. In conjunction with theoretical studies based on density functional theory (DFT) calculations, the improved photocatalytic performance of BTO‐Q was ascribed to enhanced chemisorption of nitrogen, due to formation of shallow energy levels and quantum confinement effect, as compared to the BTO‐S counterpart. To the best of our knowledge, this is the first ever demonstration of Bi_2_Ti_2_O_7_ in the photocatalytic fixation of N_2_ to NH_3_.

## 2 Results and Discussion

**Figure 1:**
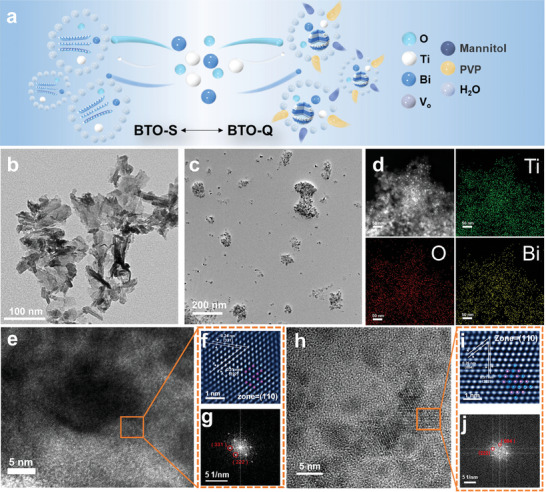
a) Schematic illustration of the preparation of BTO‐Q and BTO‐S. TEM images of b) BTO‐S and c) BTO‐Q. d) EDS‐based elemental maps of BTO‐Q. e) HRTEM image and the corresponding g) FFT and f) IFFT patterns of BTO‐S. h) HRTEM image and the corresponding j) FFT and i) IFFT patterns of BTO‐Q.

The sample preparation procedure is schematically illustrated in **Figure** **1**a, based on a one‐step hydrothermal method. For the synthesis of BTO‐Q, mannitol was added as a surfactant to limit the size of the reactors in the production of Bi_2_Ti_2_O_7_ quantum dots, and polyvinylpyrrolidone (PVP) was employed as the capping agent to protect Bi_2_Ti_2_O_7_ from being damaged by the strong reductive nature of mannitol (Figure S1, Supporting Information). Without the addition of PVP and mannitol, BTO‐S was produced instead. The details are included in Experimental Section (Supporting Information).

The crystal structures of the materials were first characterized using X‐ray diffraction (XRD) measurements. From the XRD patterns in Figure S2 (Supporting Information), both BTO‐Q and BTO‐S can be seen to exhibit a series of diffraction peaks at 2θ = 14.78, 28.51, 29.80, 34.55, 37.76, 49.66, and 58.26°, corresponding to the (111), (311), (222), (400), (331), (440), and (622) crystal planes of pyrochlore Bi_2_Ti_2_O_7_ (ICSD #180 394, Fd‐3m space group), respectively. Notably, the diffraction peaks of BTO‐Q are broader and lower in intensity as compared to those of BTO‐S, suggesting a smaller crystal grain size of the former.

In transmission electron microscopy (TEM) measurements, one can see that BTO‐S (Figure 1b) exhibited a leafy structure composed of stacked nanosheets of ca. 100 nm in length, while BTO‐Q (Figure 1c) displayed a structure consisting of aggregates of quantum dots, with an average particle diameter of ≈7.94 nm (Figure S3, Supporting Information). As the exciton Bohr radius (a_B_) of Bi_2_Ti_2_O_7_ falls in the range of 3.31–4.87 nm (i.e., 6.62–9.74 nm in diameter),^[^ ^34^ ^]^ this suggests medium‐constrained quantum confinement of the resulting BTO‐Q. In medium‐constrained quantum dots, the electron mobility is greater than that of the holes, and the electron's motion is quantized.^[^ ^35^ ^]^ Concurrently, the Coulomb interactions between electrons and holes affect the motion of holes, which move within the Coulomb field generated by restricted electrons. This allows BTO‐Q to accumulate photogenerated electrons for the activation of nitrogen gas while reducing the recombination rate of charge carriers, as manifested in the photoelectric tests that BTO‐Q exhibited a superior exciton separation efficiency and a longer lifetime of photogenerated electrons than BTO‐S (vide infra). Elemental mapping analysis based on energy‐dispersive X‐ray spectroscopy (EDS) (Figure 1d) clearly shows a consistent distribution of the Bi, Ti, and O elements across the BTO‐Q sample.

In high‐resolution TEM measurements (Figure 1e–j), BTO‐Q can be seen to exhibit well‐defined lattice fringes, with interplanar distances of 0.26 and 0.31 nm that can be assigned to the (004) and (222) crystal planes of Bi_2_Ti_2_O_7_ (ICSD #180 394), respectively (Figure 1h–j). The angle between these two crystal planes is calculated to be 54.7°, based on single crystal parameters (α = β = 90°, γ = 120°, a = b = c = 10.376 Å), which is consistent with the angle shown in Figure 1i, confirming that the growth axis of the material was along the (110) plane. BTO‐S exhibited a similar structure (Figure 1e–g), where the lattice fringes exhibited two interplanar spacings of 0.24 and 0.31 nm corresponding to the (331) and (222) planes, and the growth axis was also along the (110) plane.

**Figure 2:**
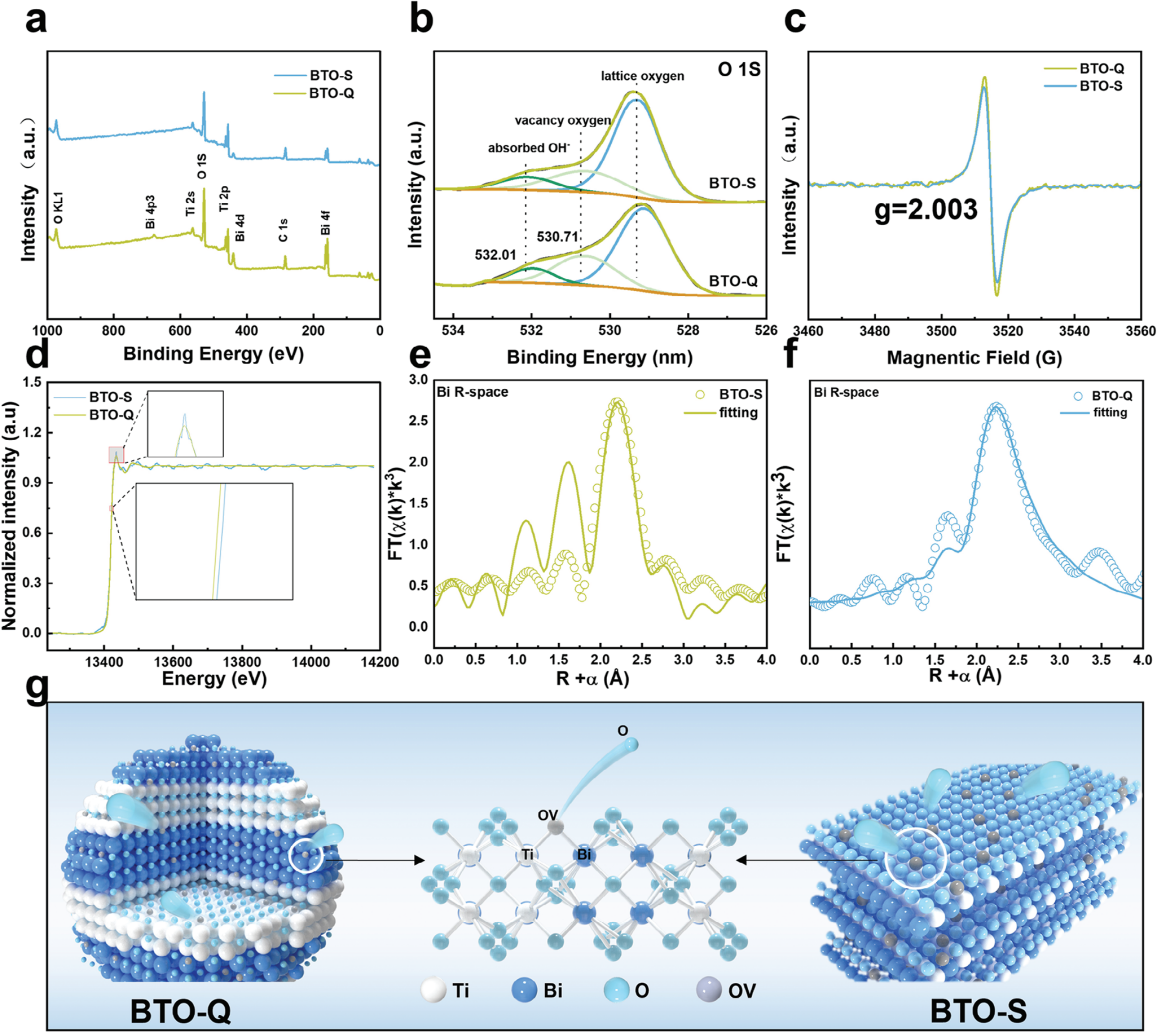
a) XPS survey spectra and b) high‐resolution scans of the O 1s electrons of BTO‐S and BTO‐Q. c) EPR spectra of BTO‐S and BTO‐Q. d) Bi L_III_‐edge XANES spectra and the corresponding k3‐weighted χ(k) function of the FT‐EXAFS spectra (insets are the zoom in of the shaded areas in the figure) and the fitting curves for e) BTO‐S and f) BTO‐Q. g) Diagram of oxygen vacancy generation sites in BTO‐S and BTO‐Q.

The surface elemental composition and valence states of the samples were then measured and compared by X‐ray photoelectron spectroscopy (XPS) measurements, with the binding energies calibrated against that of the carbon 1s peak at 284.8 eV. From the survey spectra in **Figure** **2**a, the Bi 4f, Ti 2p, and O 1s electrons can be readily discerned at ca. 163.3, 485.6, and 529.9 eV for both BTO‐S and BTO‐Q, respectively. The corresponding high‐resolution scans of the O 1s electrons for BTO‐S and BTO‐Q are shown in Figure 2b, where three peaks can be deconvoluted at 529.25, 530.74, and 532.18 eV for BTO‐S, due to lattice oxygen, oxygen vacancies, and surface hydroxyl groups, respectively.^[^ ^36^ ^]^ These three peaks shifted to a slightly lower energy for BTO‐Q at 529.12, 530.71, and 532.01 eV. From the integrated peak areas, the oxygen vacancy concentrations can be estimated to be close at 3.81% for BTO‐S and 3.95% for BTO‐Q.

Consistent results were obtained from electron paramagnetic resonance (EPR) measurements (Figure 2c), where a signal centered at ca. 3510 G (g = 2.003) can be found for both samples, confirming the generation of oxygen vacancies.^[^ ^37^ ^]^ Notably the peak‐to‐peak intensity can be seen to be only slightly greater for BTO‐Q than for BTO‐S, suggesting a similar concentration of oxygen vacancies in the two samples. This is in good agreement with the results from XPS measurements (Figure 2b).

A similar redshift is also observed with the Bi 4f and Ti 2p electrons, from 158.67 eV for Bi 4f_7/2_ of BTO‐S to 158.47 eV for BTO‐Q, and from 457.79 for Ti 2p_3/2_ to 457.61 eV (Figure S4, Supporting Information). This can be ascribed to the slightly higher concentration of oxygen vacancies in BTO‐Q than in BTO‐S (Figure 2c). Notably, these binding energies are all somewhat lower than those of pristine Bi_2_Ti_2_O_7_ (Bi 4f_7/2_ at 159.1 and Ti 2p_3/2_ at 458.4 eV),^[^ ^33^, ^38^ ^]^ in good agreement with the formation of oxygen vacancies and enhanced charge localization. Consistent results were obtained in Raman measurements (Figure S5, Supporting Information).

Both BTO‐Q and BTO‐S samples exhibited a type IV nitrogen adsorption/desorption isotherm (Figure S6, Supporting Information), from which the specific surface area was found to be markedly greater for the former (29.89 m^2^ g^−1^) than for the latter (10.33 m^2^ g^−1^), in line with their different surface morphology (Figure 1). Further structural insights were obtained from X‐ray absorption spectroscopy (XAS) measurements.^[^ ^39^ ^]^ From the Bi L_III_‐edge X‐ray absorption near edge structures (XANES) in Figure 2d (and insets), one can see that the absorption edge shifted toward a higher energy from BTO‐Q to BTO‐S, and concurrently the intensity of the white line peak increased, both indicating a higher valence state of Bi in BTO‐S.^[^ ^40^ ^]^ This is consistent with results from the above XPS measurements (Figure S4, Supporting Information). The corresponding extended X‐ray absorption fine structure (EXAFS) profiles are shown in Figure 2e–f, and the k3‐weighted fitting results are listed in Tables S1 and S2 (Supporting Information). Both BTO‐Q and BTO‐S can be seen to show a peak in the R‐space at 2.58 Å, corresponding to the Bi─O path in the Bi─O─Ti bond, with a close coordination number (CN) of ca. 0.96 for BTO‐Q and 0.98 for BTO‐S. This is in line with their similar oxygen vacancy concentrations, as manifested in the above XPS and EPR measurements. Yet the CN is markedly lower than that for pristine Bi_2_Ti_2_O_7_ (CN = 2),^[^ ^41^ ^]^ suggesting the formation of oxygen vacancies along the Bi─O─Ti path. Taken together, these observations suggest that the formation of oxygen vacancies led to electron enrichment of both the Bi and Ti sites, and the oxygen vacancies were likely situated within the Bi‐O‐Ti path (Figure 2g).

**Figure 3:**
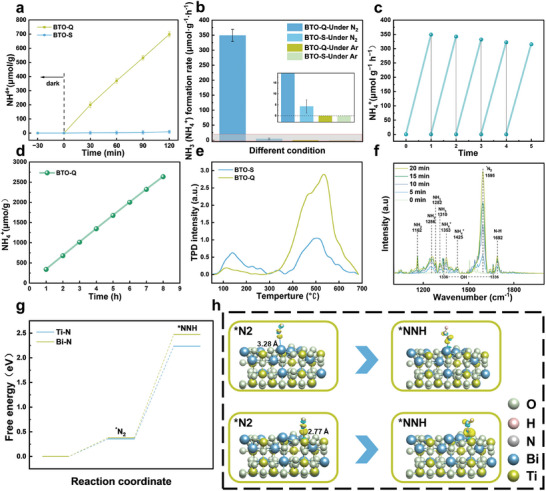
a) Quantitative determination of the generated NH_3_ under visible light irradiation catalyzed by BTO‐Q and BTO‐S. b) NH_4_^+^ synthesis rate of BTO‐S and BTO‐Q under different conditions. Inset is the zoom in of the shaded area in the figure. c) Cycling tests of photocatalytic nitrogen fixation and d) long‐term tests of BTO‐Q. e) NH_3_ TPD spectra of BTO‐Q and BTO‐S. f) In situ FTIR spectra of BTO‐Q. g) Free‐energy diagram of the nitrogen fixation process on the Bi and Ti sites of BTO. h) Calculations of charge density difference of N_2_ bonded to the Bi and Ti sites of BTO‐Q (110). Isosurface level = 0.002 e Å^−3^; negative charge, blue; positive charge, yellow.

The photocatalytic performance for nitrogen fixation to ammonia by BTO‐Q and BTO‐S was then evaluated by using a 300 W xenon lamp as the light source without the addition of co‐catalysts or sacrificial agents (Figure S7, Supporting Information), where the produced NH_4_^+^ was detected and quantified by using the Nessler reagent colorimetric method and ion chromatography (the respective calibration curves are shown in Figures S8 and S9 and Table S3, Supporting Information). The solution pH ranged from 7 to 10 in the Nessler reagent detection, and it has been shown that within this pH range, such a detection method could accurately quantify the ammonia yield.^[^ ^42^ ^]^ **Figure** **3**a shows the photocatalytic nitrogen fixation performance of BTO‐Q and BTO‐S with high‐purity nitrogen gas (99.999%) as the feed in ultrapure water by using the Nessler reagent colorimetric method. BTO‐Q can be seen to achieve an NH_3_ production of 698.18 µmol g^−1^ within 2 h at a steady‐state rate of 332.04 µmol g^−1^ h^−1^, a performance ≈77 times higher than that of BTO‐S which produced only 9.08 µmol g^−1^ of NH_3_ within 2 h at a rate of 4.29 µmol g^−1^ h^−1^. Consistent results were obtained from ion chromatography measurements (Figure S10 and Table S4, Supporting Information), where the ammonia proudction rate was estimated to be 331.87 and 4.45 µmol g^−1^ h^−1,^ respectively, confirming the reliability of the nitrogen fixation activity. Furthermore, no hydrogen gas was detected and only a small amount of oxygen was produced during the process (Figure S11, Supporting Information). This suggests excellent selectivity of BTO‐Q in nitrogen fixation. From Figure S12 (Supporting Information), it can be seen that BTO‐Q exhibited an apparent quantum efficiency (AQE) of 1.13%, 0.61%, 0.36%, and 0.12% for nitrogen production under monochromatic photoirradiation at 365, 420, 490, and 550 nm, respectively. Such a performance is superior to those of relevant catalysts reported recently in the literature (Table S5, Supporting Information). The nitrogen fixation performance of BTO‐Q and BTO‐S was also tested under an ambient atmosphere, achieving a steady‐state rate of 200.47 and 2.72 µmol g^−1^ h^−1^, respectively (Figure S13, Supporting Information). Notably, no ammonia was detected either with ambient air or high‐purity nitrogen but in the absence of photocatalysts (Figure S14, Supporting Information), confirming that the gases in the present study was free of nitrate or other impurities that could impact the nitrogen fixation performance. Note that as the oxygen vacancy concentrations were similar for both samples, the vast difference of the photocatalytic nitrogen fixation performance cannot be simply ascribed to oxygen vacancies. Therefore, further investigations were conducted to examine the photoelectric properties of the samples, as the quantum surface effect of BTO‐Q most likely led to the formation of shallow energy levels that are conducive to photocatalysis, whereas bulk‐like BTO‐S likely involved the formation of deep energy levels.

To confirm the validity of the experimental results, we conducted the photocatalytic tests under various conditions. As shown in Figure 3a,b, BTO‐Q exhibited an apparent nitrogen fixation only under visible light illumination and stable N_2_ supply, whereas no ammonia was detected in the dark or under an Ar atmosphere, indicating that ammonia was indeed produced through the photocatalytic fixation of the N_2_ feed. Furthermore, we conducted isotope labeling experiments using ^15^N_2_ as the nitrogen source. Proton nuclear magnetic resonance (^1^H NMR) measurements (Figure S15, Supporting Information) showed a doublet with a coupling constant (J_N‐H_) of 71 Hz due to ^15^NH_4_^+^. In contrast, when ^14^N_2_ was used as the gas feed, a triplet with J_N–H_ = 51 Hz that was characteristic of ^14^NH_4_^+^ was observed instead.^[^ ^43^, ^44^ ^]^ In addition, XPS measurements showed no N 1s signals in the BTO‐Q sample (Figure S16, Supporting Information). Taken together, these results confirmed the absence of exogenous nitrogen sources and the NH_4_^+^ detected was produced solely from the N_2_ feed.

Furthermore, we tested the stability of BTO‐Q. After five cycles of experiments of the same batch of sample, the material retained ≈90% of the initial performance (Figure 3c); and after 8 h's continuous testing, the ammonia production rate remained virtually unchanged (Figure 3d). TEM and XRD measurements did not reveal significant structural changes in the BTO‐Q sample (Figures S17 and S18, Supporting Information). No apparent variation was observed of the oxygen vacancies either, as manifested in XPS and EPR measurements (Figures S19 and S20, Supporting Information). These suggest high stability of the BTO‐Q sample. Consistent results were obtained from ab initio molecular dynamics (AIMD) simulations. Note that whereas Kinetic Monte Carlo (KMC) and AIMD represent the leading computational methods of studying the stability of materials, and the KMC method is advantageous in observing macroscopic dynamics^[^ ^45^ ^]^ and is cost‐effective,^[^ ^46^ ^]^ it struggles to provide accurate structural and energy information. Therefore, we opted to use AIMD to examine the stability of the BTO samples, where the bond lengths between the titanium active sites adjacent to oxygen vacancies and neighboring atoms were monitored in real time on the BTO (110) surface (Figures S21 and S22, Supporting Information). Within a dynamic time scale of 5000 fs, virtually no distortion of the Ti─O bonds (i.e., O_1_, O_2_, O_3_, and O_4_ in Figure S21, Supporting Information) was observed, and no bond breaking event was detected in snapshots of the dynamic simulation (Figure S22, Supporting Information). This indicates moderate stability of BTO grown along the (110) plane.

Temperature‐programmed desorption of nitrogen (N_2_‐TPD) was then employed to investigate the N_2_ adsorption properties on the surfaces of BTO‐S and BTO‐Q (Figure 3e). For BTO‐S, a single desorption peak started at 86 °C and centered at ca. 140 °C, indicative of physical adsorption of N_2_. In contrast, N_2_ desorption on BTO‐Q commenced at a higher temperature (ca. 338 °C), with two peaks observed at 465.25 and 536.68 °C, suggestive of chemical adsorption of N_2_ on the sample surface. This indicates that physical adsorption of N_2_ was dominant on bulk‐like BTO‐S, whereas BTO‐Q facilitated chemical adsorption. As chemical adsorption of N_2_ molecules is a crucial step for the activation and eventual reduction of N_2_, this most likely played a critical role in enhancing the photocatalytic nitrogen fixation performance, as observed above.

In situ infrared spectroscopy was employed to further investigate the adsorption, activation, and hydrogenation of nitrogen on the surface of BTO‐Q. As shown in Figure 3f, under illumination, the vibrational peak of chemisorbed N_2_ molecules (^*^N_2_) at 1595 cm^−1^ gradually intensified, indicating enhanced chemical adsorption of N_2_ on the surface of BTO‐Q, consistent with the TPD experimental results.^[^ ^47^ ^]^ Meanwhile, an increase of the vibrational band intensity was also observed for the peaks at 1162, 1256, and 1310 cm^−1^ due to the characteristic vibrations of adsorbed NH_3_, 1353 and 1425 cm^−1^ due to surface NH_4_^+^,^[^ ^48^ ^]^ and 1282 and 1692 cm^−1^ due to NH_2_ and NH intermediates.^[^ ^49^ ^]^ This indicates enhanced conversion of N_2_ molecules to NH_4_^+^ by prolonged illumination. The vibrational bands at 1336 and 1663 cm^−1^ can be attributed to the OH species. It is noteworthy that characteristic vibrations of N_2_H_4_ were not observed at 1129 and 1290 cm^−1^, further confirming the selective production of NH_4_^+^ from nitrogen fixation (Figure S23, Supporting Information).^[^ ^8^, ^50^ ^]^

To delve deeper into the mechanism of the nitrogen reduction reaction, DFT calculations were conducted. Based on the experimental results in Figure 1, a BTO(110) slab was constructed as the structural model. The primary location of oxygen vacancies was determined to be at the O connected to Bi and Ti (Bi–O–Ti). It was found that during the end‐on adsorption of N_2_ molecules onto the Bi sites (Figure S24, Supporting Information), the distance between Bi and N is ca. 3.28 Å, and the adsorption is relatively weak. However, when N_2_ molecules adsorb onto the Ti sites, the distance between Ti and N is shortened to 2.77 Å, suggesting that Ti is energetically favored as the adsorption sites. Additionally, the Gibbs free energy for N_2_ fixation and hydrogenation at both Bi and Ti sites is shown in Figure 3g, where the N_2_ adsorption energy at the Bi site (0.382 eV) is higher than that at the Ti site (0.357 eV). In the process of N_2_ reduction to NH_3_, the first hydrogenation step involves the protonation of N_2_ to form N‐NH (N_2_ + H^+^ → N‐NH), which is a rate‐limiting step in the entire N_2_ fixation process. At the Bi site, this requires an energy of 2.089 eV, while only 1.879 eV at the Ti site, implying that Ti atoms play a primary role in the adsorption and activation of nitrogen on BTO‐Q. In the calculation of charge density differences, a significant electron depletion is observed at the Ti atoms and electron accumulation around the N atoms during the nitrogen fixation and hydrogenation processes (Figure 3h). This suggests electron transfer from Ti to N, which weakens the N─N bond, whether on the N_2_ or NNH stage. In contrast, when N_2_ is adsorbed onto Bi atoms, there is almost no observable electron depletion or accumulation around the N atoms. This result is consistent with the Gibbs free energy calculations, indicating a negligible role of Bi in the reaction, and Ti most likely serves as the active sites in the reaction.

Additionally, as hydrogen evolution reaction (HER) is a well‐known competitive reaction in photocatalytic nitrogen fixation, we also calculated the surface adsorption energy of H on the model surface (Figure S25, Supporting Information).^[^ ^51^, ^52^ ^]^ The results show that when H adsorbs onto the Ti atom, the Gibbs adsorption energy is 0.425 eV, much higher than that of N_2_ (0.357 eV). A similar behavior is observed on the Bi site. This suggests minimal interference of HER in the fixation and activation of N_2_ on BTO, consistent with the high selectivity for nitrogen fixation observed experimentally.

**Figure 4:**
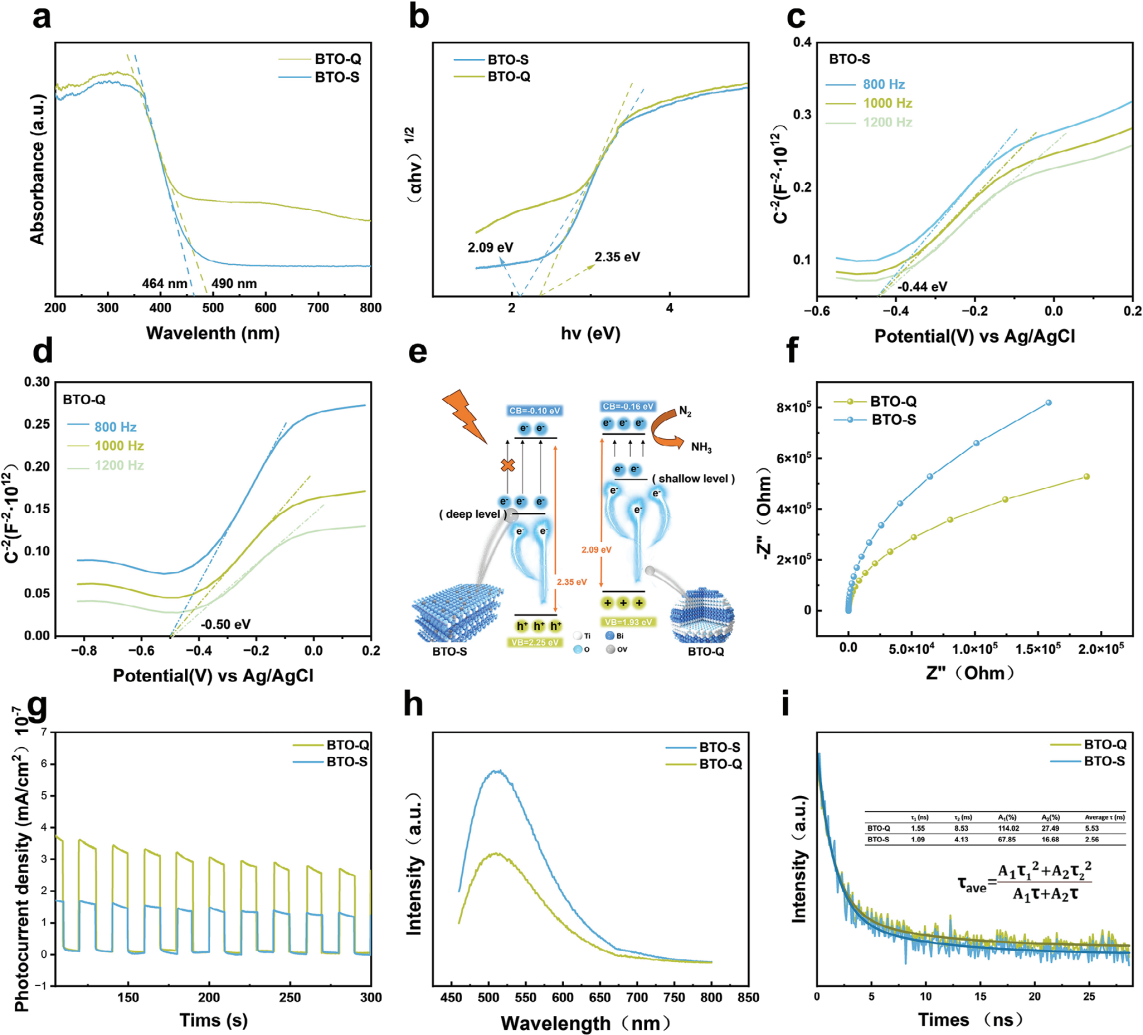
a) UV–vis DRS of the samples, and b) the corresponding plots of transformed Kubelka–Munk functions versus photon energy to estimate bandgaps for BTO‐S and BTO‐Q. Mott–Schottky plots of c) BTO‐S and d) BTO‐Q. e) Bandgap diagrams for BTO‐S and BTO‐Q. f) Nyquist plots of BTO‐S and BTO‐Q. g) Photocurrent curves of BTO‐S and BTO‐Q at various times under solar‐light irradiation using a three‐electrode setup. h) Steady‐state and i) time‐resolved PL emission spectra of BTO‐S and BTO‐Q. Excitation wavelength 445 nm. Inset lists the τ values obtained from the bi‐exponential fitting of the data.

**Figure** **4**a shows the UV–vis DRS profiles of the prepared samples. The absorption edge of BTO‐S was estimated to be ≈464 nm, while 490 nm for BTO‐Q, with the characteristic continuous attenuation absorption tail associated with oxygen vacancies. Additionally, the band structures of BTO‐S and BTO‐Q were determined from the Tauc plots (Figure 4b) and Mott–Schottky profiles (Figure 4c,d). The bandgap (E_g_) of the samples can be calculated using the Kubelka–Munk equation, αhν = A(hν − E_g_)^n/2^. The value of n depends on the type of semiconductor transition, and for indirect semiconductors like BTO, n can be set at 4.^[^ ^12^, ^53^ ^]^ Calculations based on this assumption yield a bandgap of 2.35 eV for BTO‐S and somewhat lower at 2.09 eV for BTO‐Q. The flat‐band potential was then assessed from the Mott–Schottky profiles at multiple frequencies, −0.44 eV for BTO‐S and −0.50 eV for BTO‐Q (Figure 4e).

The reduced bandgap of BTO‐Q suggested enhanced light absorption compared to BTO‐S. For quantum dots with a size smaller than the exciton Bohr radius, i.e., strong‐confinement quantum dots, the curvature and kinetic energy of the wavefunction increase, and the kinetic energy dominates the Coulomb interaction, causing a blue shift in the bandgap due to the confinement of charge carriers in a reduced space.^[^ ^54^, ^55^ ^]^ BTO‐Q, being a moderately constrained quantum dot, does not exhibit a pronounced constraint effect on charge carriers, and as a result, no significant blue shift in the band gap occurs. It must be emphasized that when oxygen vacancies are formed in a semiconductor, additional energy levels are produced, thereby affecting the band gap energy of the semiconductor. The introduced energy levels can be classified as shallow levels and deep levels, with shallow levels having a more significant impact on the band gap and optical properties than deep levels.^[^ ^32^, ^56^ ^]^ It is noteworthy that in BTO‐Q, both the clearly discernible continuous decay of the DRS absorption tail and the narrowing of the band gap indicate the formation of shallow energy levels associated with oxygen vacancies. By contrast, oxygen vacancies produce primarily deep energy levels in bulk‐like BTO‐S. In comparison to the broadening of the band gap in strongly confined quantum dots, the observed changes in the band gap and optical absorption capacity in BTO‐Q are evidently advantageous for photocatalytic nitrogen fixation, in good alignment with the experimental results.

Mechanistically, when defect energy levels within the bandgap shift, the Fermi level (E_f_) moves toward the defect levels to balance the additional electronic states.^[^ ^57^ ^]^ This was indeed observed in ultraviolet photoelectron spectroscopy (UPS) measurements. From Figure S26a,b (Supporting Information), one can see that the work functions (Φ) of BTO‐S and BTO‐Q are at 4.39 and 3.78 eV, respectively (vs vacuum level). In conjunction with results from the Tauc plots, we can deduce that the difference between E_f_ and the valence band maximum (VBM) was 1.45 eV for BTO‐S and 1.81 eV for BTO‐Q. The fact that the E_f_ of BTO‐Q is closer to the conduction band also confirms the change in defect energy levels (Figure S26c, Supporting Information). Therefore, in the photocatalytic synthesis of ammonia, moderately confined quantum dots are more favorable for the enhanced performance, due to the “activation” of oxygen vacancies in the formation of shallow energy levels, as demonstrated above.

Electrochemical impedance measurements (Figure 4f) showed a smaller semicircle radius for BTO‐Q than for BTO‐S, suggesting a lower charge‐transport resistance (R_CT_). Consistently, the photocurrent density of BTO‐Q was higher than that of BTO‐S (Figure 4g). These observations indicate that photo energy was more effectively converted into free carriers (electrons and holes) on BTO‐Q. That is, under photo illumination, more photo‐excited electrons were accumulated on BTO‐Q than on BTO‐S, demonstrating a higher electron‐hole separation efficiency. Notably, the sample appearance was virtually unchanged before and after the electrochemical tests, demonstrating high stability of the sample and reliability of the results (Figure S27, Supporting Information). These observations suggest that with the increased surface area of the quantum dots, oxygen vacancies facilitate the formation of shallow energy levels within the band gap. Concurrently, the quantum size effect makes it easier for photo‐generated electrons to migrate to the material surface. Additionally, surface oxygen vacancy defects are conducive to electron accumulation, thereby reducing the recombination of photo‐generated electrons and vacancies.

Consistent results were obtained from PL spectroscopic measurements. From Figure 4h, one can see that the intensity of the steady‐state PL emission of BTO‐Q was lower than that of BTO‐S. Further studies were conducted with time‐resolved PL spectroscopic measurements (Figure 4i). Note that the PL lifetime of quantum dots typically consists of two parts, a long lifetime (τ_1_) resulting from the slow decay of trap states caused by surface effects (such as surface defects, vacancies, and impurities), and a short lifetime (τ_2_) originating from direct transitions radiating from the conduction band bottom to the valence band, representing the emission of band‐edge excitonic states.^[^ ^58^, ^59^ ^]^ It can be observed that the exciton long lifetimes (τ_1_) for BTO‐S and BTO‐Q are rather close at 1.09 and 1.55 ns (Figure 4i inset; Table S6, Supporting Information), respectively, consistent with a relatively small difference in defect concentration in the samples (Figure 2). Additionally, the short lifetimes (τ_2_) for BTO‐S and BTO‐Q are 4.13 ns and 8.53 ns, respectively, resulting in an average lifetime (τ_ave_) of 2.56 and 5.53 ns. This suggests that BTO‐Q possessed a slower exciton recombination rate, in good agreement with the variation observed in the photocatalytic nitrogen fixation performance. These results further confirm the enhanced charge carrier separation efficiency and reduced recombination capability of nanoscale BTO‐Q as compared to bulk‐like BTO‐S under light irradiation.

## 3 Conclusion

In this study, pyrochlore Bi_2_Ti_2_O_7_ quantum dots and nanosheets were prepared by a one‐pot hydrothermal procedure. Although both samples exhibited a similar level of oxygen vacancies, the photocatalytic performance of BTO‐Q for ammonia fixation was significantly enhanced. The steady‐state rate of nitrogen fixation on BTO‐Q under a nitrogen atmosphere was 332.04 µmol g^−1^ h^−1^, which was 77 times higher than that of BTO‐S. Experimental and DFT studies showed that the shallow energy levels arising from oxygen vacancies in the quantum dots, in conjunction with the medium‐constrained quantum confinement effect, were responsible for the enhanced mobility and separation of photogenerated charge carriers, and hence adsorption and activation of nitrogen molecules. Meanwhile, results from ab initio molecular dynamics simulation suggest remarkable structural stability of the BTO samples; and further mechanistic insights were obtained from DFT calculations where minimal HER interference was suggested, in good agreement with the high selectivity of nitrogen fixation observed experimentally. Results from this work highlight the fundamental significance of the synergy of size confinement and defect engineering in the development of high‐efficiency photocatalysts toward nitrogen fixation.

## Conflict of Interest

The authors declare no conflict of interest.

## Supporting information



Supporting Information

## Data Availability

The data that support the findings of this study are available from the corresponding author upon reasonable request.
