# Supplementary material for: Bi2Ti2O7 Quantum Dots for Efficient Photocatalytic Fixation of Nitrogen to Ammonia: Impacts of Shallow Energy Levels
Source: Adv Sci (Weinh). 2024 Sep 5;11(41):2408829. doi: 10.1002/advs.202408829 (PMC11538629; doi:10.1002/advs.202408829)
Supplement: Supplementary file 1 — Supporting Information [file ADVS-11-2408829-s001.pdf]

ADVANCED SCIENCE
Open Access

Supporting Information

for *Adv. Sci.*, DOI 10.1002/advs.202408829

$Bi_2Ti_2O_7$ Quantum Dots for Efficient Photocatalytic Fixation of Nitrogen to Ammonia: Impacts of Shallow Energy Levels

Pengkun Li, Runjie Wu, Peishen Li, Shuai Gao, Zeping Qin, Xingjian Song, Wenming Sun, Zhaorui Hua, Qiang Wang* and Shaowei Chen**

Supporting Information

$Bi_2Ti_2O_7$ quantum dots for efficient photocatalytic nitrogen fixation to ammonia: Impacts of shallow energy levels

Pengkun Li [a], Runjie Wu [a], Peishen Li [c], Shuai Gao [a], Zeping Qin [a], Xingjian Song [b], Wenming Sun [a,*], Zhaorui Hua [a], Qiang Wang [a,*] and Shaowei Chen [b,*]

[a] Laboratory for Micro-sized Functional Materials & College of Elementary Education and Department of Chemistry, Capital Normal University, Beijing 100048, China
[b] Department of Chemistry and Biochemistry, University of California, 1156 High Street, Santa Cruz, California 95064, United States
[c] College of Environmental Sciences and Engineering, Key Laboratory of Water and Sediment Sciences (MOE), Peking University, Beijing, 100871, China

* E-mail: shaowei@ucsc.edu (S.W.C.), gwchem@gmail.com (Q.W.)

List of Contents

- Experimental details
- 27 figures
- 6 tables

Experimental details

Materials

All chemicals were of analytical purity. purchased from Shanghai Macklin Biochemical Co., Ltd and used without further purification. Ultrapure water was used in all experiments.

Synthesis of Bi_2TiO_7 quantum dots

In a typical experiment, 4 mmol of bismuth nitrate was dissolved into 10 mL of dilute nitric acid. Separately, 8 mmol of titanium sulfate was dissolved in ultrapure water. These two solutions were then mixed, into which was added 7 mmol of PVP and 6 mmol of mannitol under sonication for 30 min to obtain a milky white solution, where the solution pH was adjusted to ca. 12 by the addition of NaOH. The resultant mixture was transferred to a Teflon-lined auto clave (50 mL) and hydrothermally treated at 200 ^{o}C for 12 h. After the reaction, the samples were collected by centrifugation and rinsed with a copious amount of ethanol and water. The obtained sample was denoted as BTO-Q.

Synthesis of Bi_2TiO_7 sheets

BTO-S was prepared in the same fashion except for the addition of PVP and mannitol.

Structural characterizations

The microstructure and morphology of the photocatalysts were examined by scanning electron microscopy measurements (SEM, S-4800 Hitachi, Japan). Transmission electron microscopic (TEM) and high-resolution transmission electron microscopic (HRTEM) measurements were performed with a JEM-2010 electron microscope (Japan). The phase structure of the samples was probed by X-ray diffraction (XRD) measurements with a Shimadzu X-ray diffractometer (XRD-6100) using Cu Kα radiation. X-ray photoelectron spectroscopy (XPS) measurements were performed with a Thermo Kratos Axis Supra instrument. All binding energies were calibrated against the C 1s peak at 284.8 eV. X-ray absorption (XAS) data were collected at the Shanghai Synchrotron Radiation Facilities. A Thermo UV-2600 UV-visible spectrophotometer was used to acquire the photo absorption and UV-vis diffuse reflectance spectroscopy (DRS) profiles. Photoluminescence (PL) measurements were performed with an Edinburgh FLS9 fluorescence spectrometer. Fourier transform infrared (FTIR) spectra were acquired at room temperature with a Bruker tensor II FTIR NEXUS spectrometer in the frequency range of 800 to 4000 cm^{-1}. Raman spectra were recorded at room temperature using a Horiba miniature Raman spectrometer in a backscattered geometry with a 532 nm laser as the excitation source. Brunauer-Emmett-Teller (BET) surface area was analyzed with a nitrogen sorption device (BET, Mike ASAP2020). Electron paramagnetic resonance (EPR) spectra were collected with a Bruker A200 spectrometer. The EPR spectra of SBTO-2 before and after photocatalytic nitrogen reduction reaction were acquired with an A300 instrument. Generation of oxygen during photocatalytic nitrogen fixation was detected with a dissolved oxygen meter (JPSJ-660L), where the dissolved oxygen content (DOC) in the reaction solution was recorded in 30 min for light-driven catalytic systems under a nitrogen atmosphere. Ion chromatography was used to detect NH_4^+ with a ThermoFisher Scientific-Aquion instrument. Nitrogen temperature programmed desorption (N_2-TPD) was conducted on a Tianjin, China, Xianquan TP5080 instrument. Ultraviolet photoelectron spectroscopy (UPS) measurements were conducted using a ThermoFisher Nexsa instrument.

Photocatalytic nitrogen fixation

A 300 W Xe lamp was used as the UV-visible light source and a closed single gas channel N_2 photocatalytic reactor was used as the vessel for fresh NH_4^+ production (Figure S8). The light source was located 15 cm away from the surface of the photocatalyst suspension and provided an intensity of

about 2 suns (200 mW·cm^{-2}). Typically, 20 mg of a photocatalyst was dispersed under stirring into 100 mL of ultrapure water. In the dark, high-purity N_2 (99.999%, with ≤ 3 ppm O_2, ≤ 1 ppm H_2, ≤ 3 ppm THC, and ≤ 5 ppm moisture) was passed continuously at a rate of 120 mL min^{-1} for 1 h to create a pure N_2 environment (positive pressure). The light source was then turned on and an aliquot (5 mL) of the reaction solution was removed every 30 min during the photocatalytic reaction and filtered with a 0.22 μm filter tip. Subsequently, NH_4^+ was quantified using both ion chromatography and the Nessler's reagent method. The specific steps for the Nessler's reagent method are described below.

Quantitative analysis of NH_4^+ concentration

The NH_4^+ content generated during the photocatalytic nitrogen fixation process was quantified by Nessler's reagent. First, a series of NH_4Cl solutions at different concentrations were prepared, into which was then added potassium sodium tartrate ($KNaC_4H_4O_6$, 0.1 mL, 0.5 mg L^{-1}), followed by Nessler's reagent (0.1 mL) for 10 min's color development. The absorbance at 420 nm was recorded with 0 mg L^{-1} NH_4Cl as the blank calibration, and the standard curve was plotted with NH_4^+ concentration as the x axis and absorbance as the y axis. Ammonia concentration was also quantified using a ThermoFisher AQUION ion chromatograph (IC).

Calculation of exciton Bohr radius

The exciton Bohr radius is calculated by using the equation, $\mathbf{log(a_{B,ex})}$ = a + b log(E_g), where $a_{B,ex}$ is the exciton Bohr radius expressed in nanometers, E_g is the bulk energy bandgap at 300 K expressed in electron volts, a = 1.04434 ± 0.04976, and b = -1.37696 ± 0.10488.[1]

Electrochemical measurements

Photocurrent response and electrochemical impedance spectroscopy measurements were performed in a three-electrode configuration (CHI 760E, Shanghai, China), where 0.1 M Na_2SO_4 was used as the electrolyte solution, a Pt sheet as the counter electrode, and a saturated calomel electrode (SCE) as the reference electrode. The light source was turned on during the acquisition of the electrochemical impedance data. The working electrode was prepared by adding 5 mg of the above prepared samples in 0.125 mL of ethanol, 0.375 mL of ultrapure water and 50 μL of Nafion under sonication and dropcast onto the surface of an FTO conductive glass electrode with an area of 1 cm x 4 cm.

Calculation of apparent quantum efficiency

The apparent quantum efficiency (AQE) was determined by using 365, 420, 490 and 550 nm monochromatic light sources, with the following equation:

$$\text{AQE}=\frac{Ne}{Np}=\mathbf{3}\times\boldsymbol{n(NH_4^+,mol)}\times\frac{N_A}{w\times\frac{t}{hv}}$$

where $\boldsymbol{N_e}$ is the total number of electron transfer, $\boldsymbol{N_p}$ is the number of incident photons, $\boldsymbol{n}$ ($\boldsymbol{NH_4^+}$, $\boldsymbol{mol}$) is the amount of $\mathbf{NH_4^+}$ produce, $\boldsymbol{N_A}$ is Avogadro's constant, h is Planck's constant, **W** is the total incident light energy (incident light intensity × irradiation area), **t** is the irradiation time of incident light (s), and ν is the frequency of the incident light. The incident light intensity is 10 mW cm^{-2}, and the irradiation area is 6.28 cm^2.

Performance tests of water decomposition

In a typical nitrogen reduction experiment, gases produced from the reaction were collected every hour and the compositions were analyzed with a Labsolar-6A (Beijing, China-Bofeilai) gas chromatography instrument.

Computational details

All the calculations were implemented with density functional theory (DFT) method via Quantum Espresso package.[2, 3]The revised Perdew-Burke-Ernzerhof (PBEsol)[4] functional in the generalized gradient approximation (GGA) was adopted to describe the exchange-correlation function. SSSP pseudopotential libraries (precision version)[5] were selected as suggested by N. Marzari et al[6]. DFT-D3 scheme was utilized to consider the van der Waals interaction.[7] Kinetic energy and charge density cutoffs cutoff of 30 Ry and 300 Ry with a Marzari–Vanderbilt cold smearing[8] width of 0.04 eV were selected in the geometrical optimization.

The calculated lattice constants of $Bi_2Ti_2O_7$ are a = b = c = 9.972 Å, in good agreement with the experimental results.[9] According to the HRTEM results, a clean (110) surface and an OV-containing (110) surface were constructed to consider the adsorption configurations of both hydrogen atom and N_2 molecule. The adsorption Gibbs energy was calculated based on the computational hydrogen electrode (CHE) model.[10] The change in free energy (ΔG) for the considered steps was calculated using the following equation $\Delta G = \Delta E + \Delta ZPE - T\Delta S$, where ΔE, ΔZPE, and ΔS are the differences in the total energy, zero-point energy, and entropy between the reactant and product, respectively. The ΔZPE and ΔS values reported by Liu et al. were adopted.[11]

To prevent erroneous interactions between neighboring periodic images, a vacuum layer of 15 Å along the z-axis was added. Monkhorst–Pack k-point grids were 2 × 2 × 1 in Brillouin zone sampling. Geometry optimization was performed until the maximum residual force was 0.03 eV $Å^{-1}$. The convergence requirement was chosen at 1×10^{-6} eV $atom^{-1}$ for SCF iterations.

Ab initio molecular dynamics simulations were performed to investigate the stability of substrates. To make a balance between computing speed and convergence accuracy, an energy cutoff was set at 350 eV, the SCF tolerance and Brillouin zone sampling were set at 1×10^{-5} eV $atom^{-1}$ and 1 × 1 × 1, respectively. The simulation was carried out at constant volume and temperature (NVT), with the temperature controlled using a Nose-Hoover thermostat. The simulation temperature was fixed at 298 K and a sufficient simulation of 5 ps with a time step of 1 fs was chosen for the calculation.

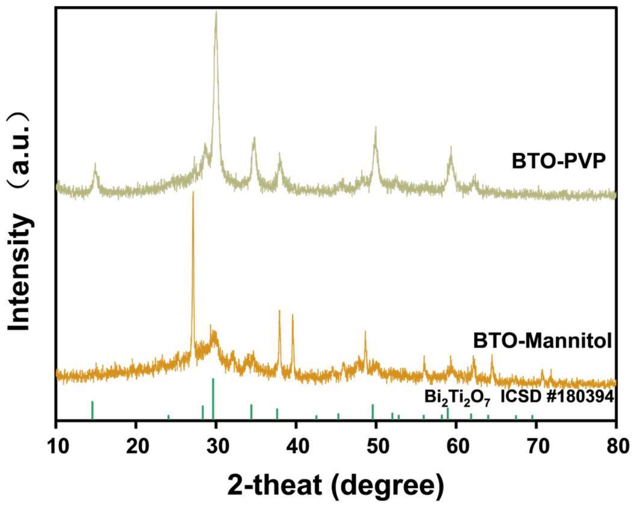

Figure S1. BTO samples treated separately with PVP and mannitol.

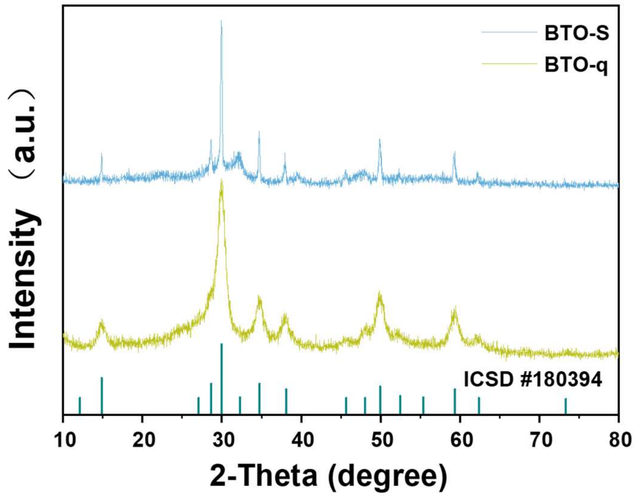

Figure S2. XRD patterns of BTO-Q and BTO-S.

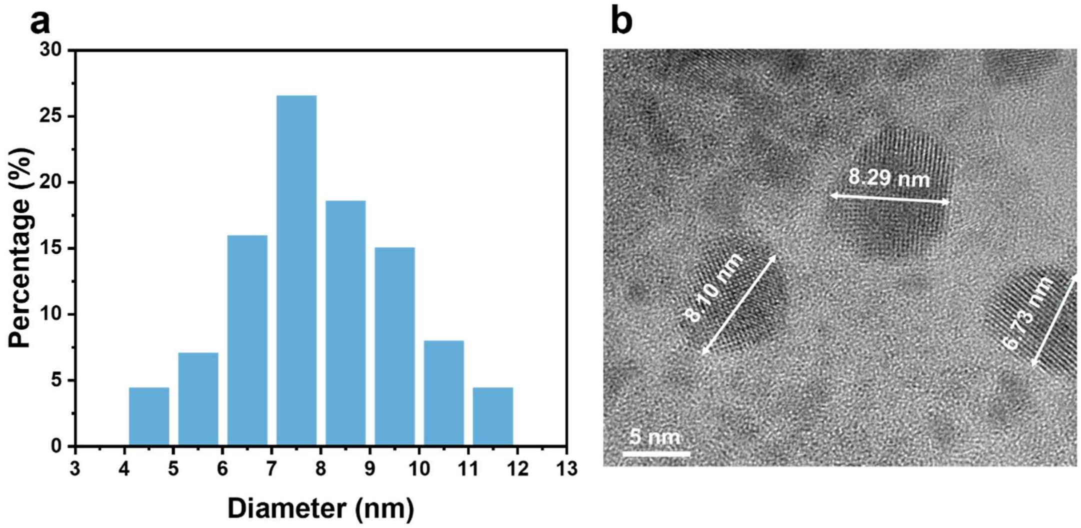

Figure S3. (a) Particle size histogram of BTO-Q. (b) High-resolution TEM image of BTO-Q.

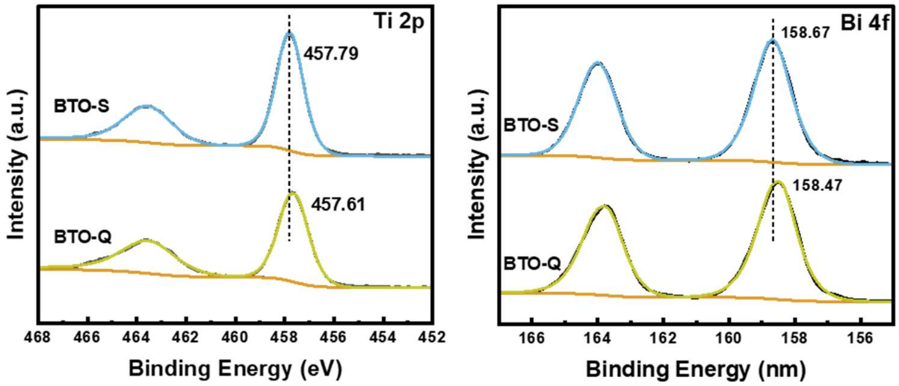

Figure S4. High-resolution scans of the Ti 2p and Bi 4f electrons of BTO-S and BTO-S.

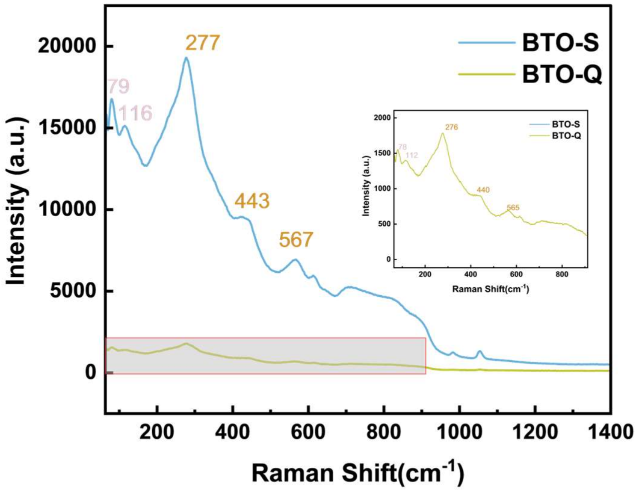

Figure S5. Raman spectra of BTO-S and BTO-Q. Inset is the zoom in of the shaded box of the BTO-Q sample.

From the figure, one can see that BTO-S exhibits three distinct peaks at 277 cm^{-1} (E_g), 443 cm^{-1} (F_{2g}), and 567 cm^{-1} (A_{1g}), attributed to the stretching vibrations of Ti-O. Additionally, peaks at 79 cm^{-1} (F_{1u}) and 116 cm^{-1} (F_{2g}) correspond to the bending vibrations of Bi-O-Bi and Bi-O.[12] It is worth noting that in the Raman spectrum of BTO-Q, the peaks for Bi-O and Ti-O are slightly blue-shifted, as compared to BTO-S, consistent with the assumption of oxygen vacancies located in Bi-O-Ti. Meanwhile, the Raman spectrum of BTO-Q shows lower intensity, consistent with the performance of quantum dots in Raman spectra.[13]

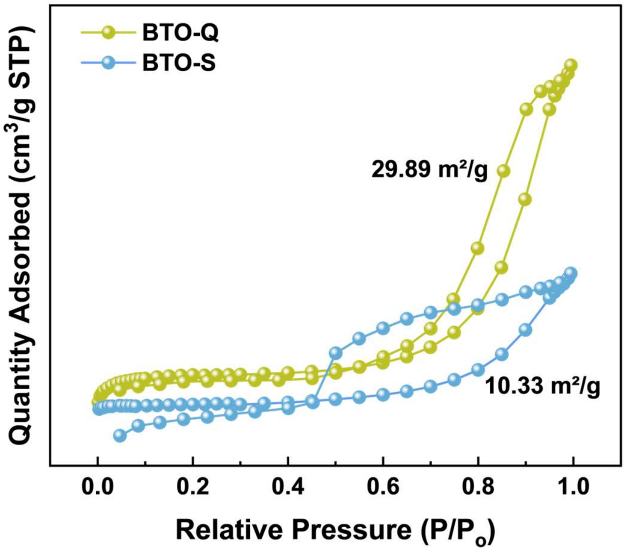

Figure S6. N_2 adsorption–desorption isotherms of BTO-S and BTO-Q.

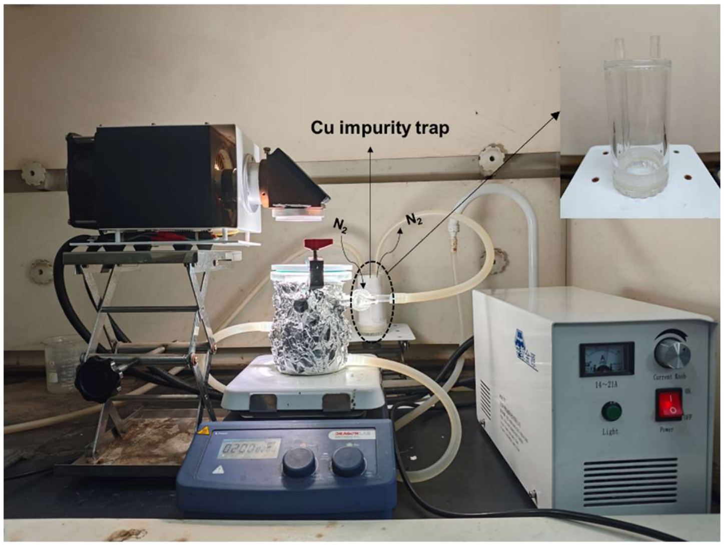

Figure S7. Photograph of the set up for photocatalytic nitrogen fixation experiments. Inset shows the N2 purification setup where a copper catalyst (Cu-SSZ-13) is used as an impurity trap to purify the gas. Numerous studies have shown that this is an effective method for eliminating NOx.[14-16]

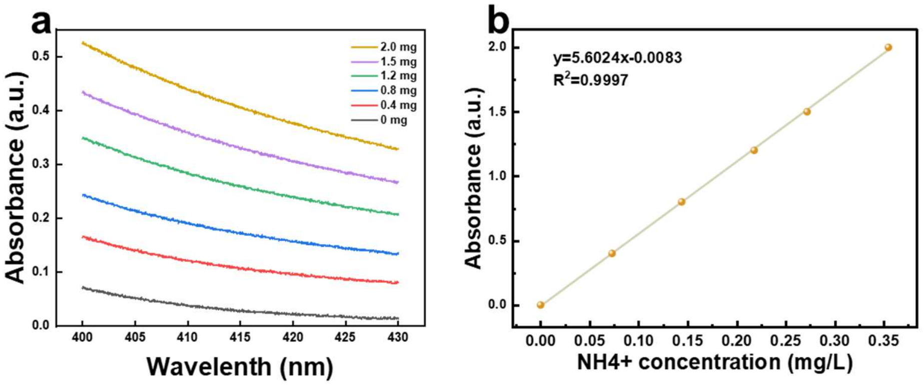

Figure S8. (a) UV-vis absorption spectra of Nessler's colorimetric method assays with NH_4Cl at different concentrations, and (b) the calibration curve for NH_4Cl.

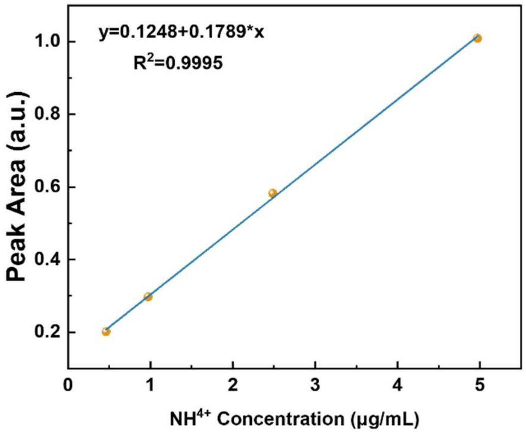

Figure S9. Standard working curve of the concentration of NH_4^+ by ion chromatography.

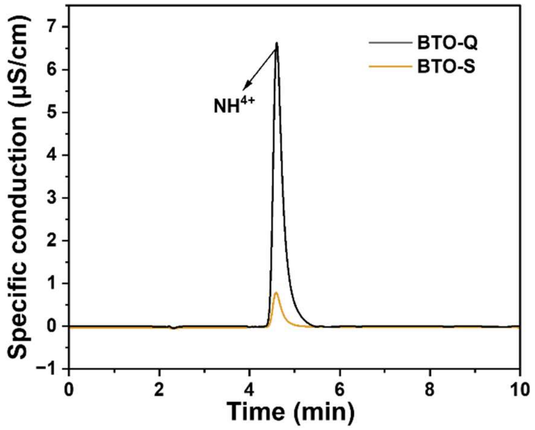

Figure S10. Ammonia concentration detected by ion chromatography by photocatalytic nitrogen fixation with BTO-Q and BTO-S for 2 h.

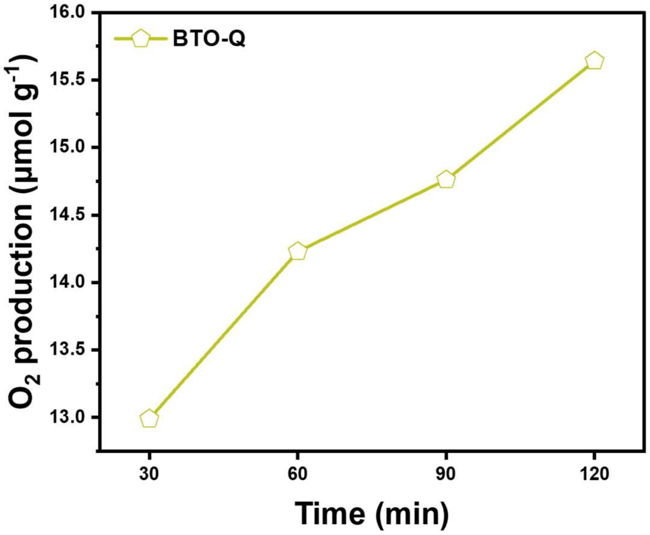

Figure S11. Oxygen production in photocatalytic nitrogen fixation by BTO-Q.

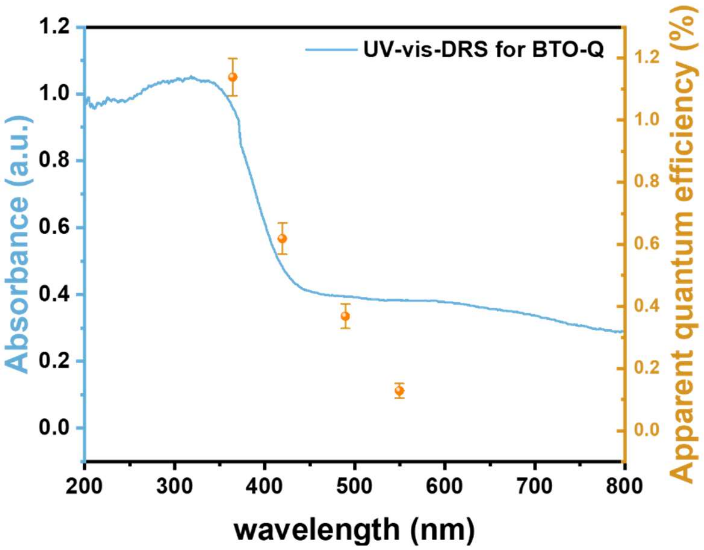

Figure S12. Wavelength-dependent AQE of ammonia production (right axis) and UV-vis absorption spectrum (left axis) for BTO-Q.

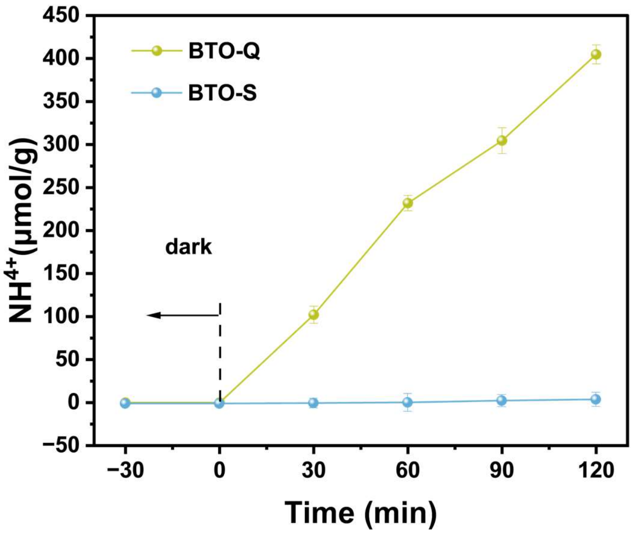

Figure S13. Quantitative measurement of NH_3 generation under visible light for BTO-Q and BTO-S in ambient air atmosphere.

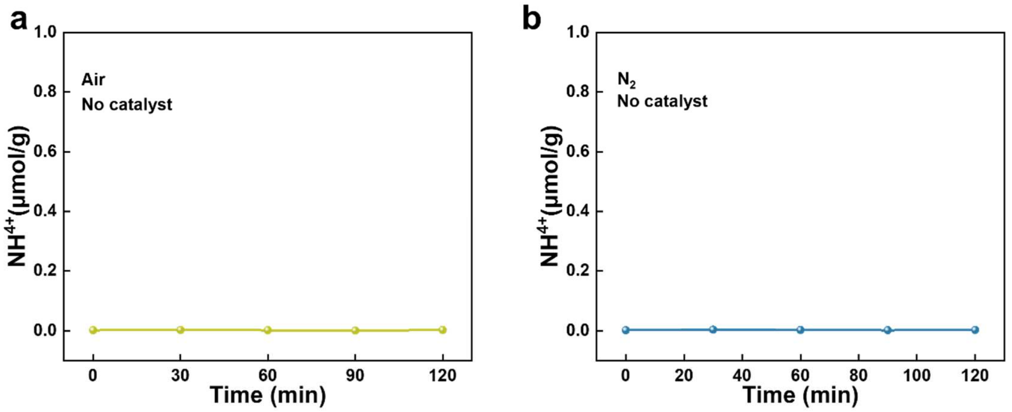

Figure S14. Ammonia content after the continuous feed of (a) air and (b) high-purity nitrogen into the reactor in the absence of a catalyst.

One can see that when ambient air or high-purify nitrogen was fed into ultrapure water, essentially no ammonia was produced in the absence of photocatalysts. This suggests that the gases used in the present study did not contain nitrate or other impurities that could impact the nitrogen fixation performance.

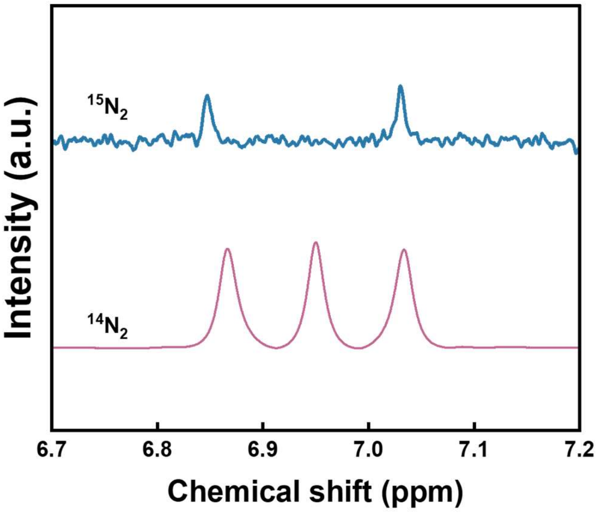

Figure S15. ^{1}H NMR spectra of standard $^{14}NH_4Cl$ and the fixation product from a $^{15}N_2$ feed.

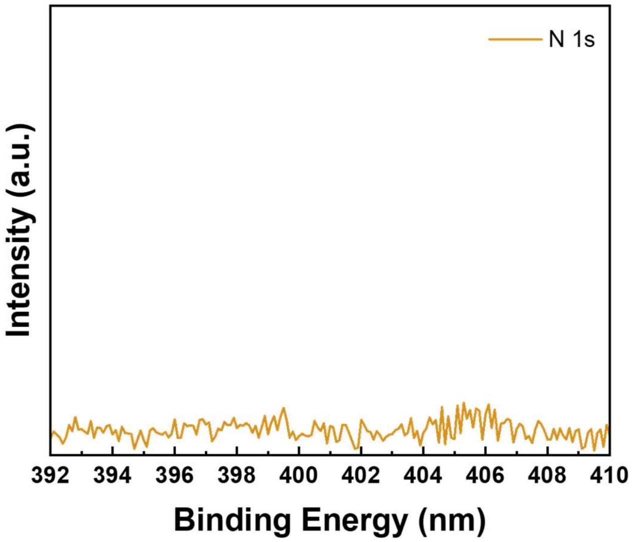

Figure S16. High-resolution scan of the N 1s electrons of BTO-Q.

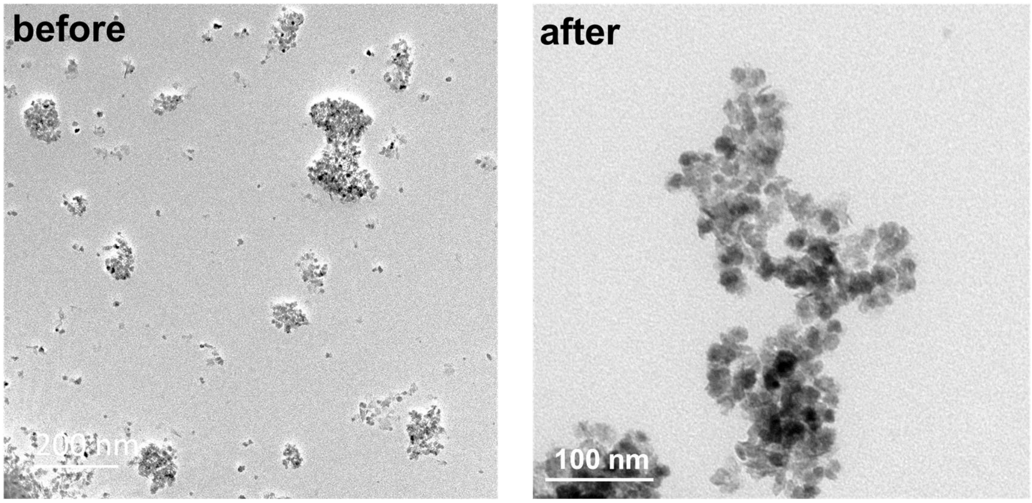

Figure S17. TEM images of BTO-Q before and after photocatalytic nitrogen fixation reaction

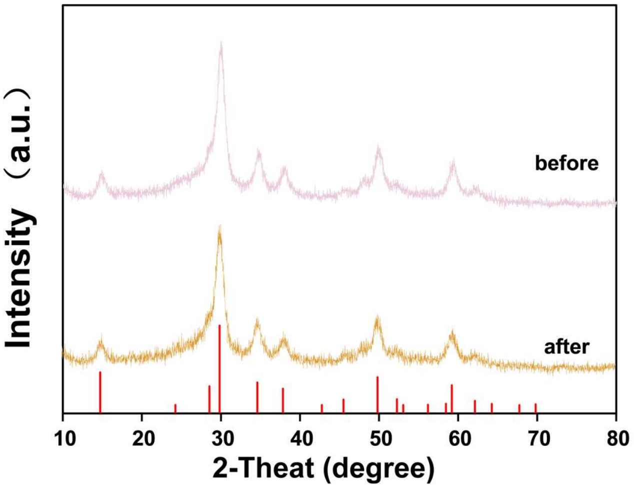

Figure S18. XRD patterns of BTO-Q before and after photocatalytic nitrogen fixation reaction.

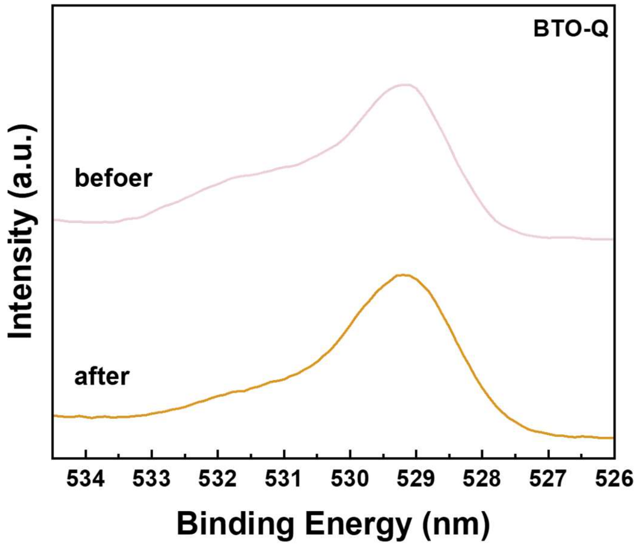

Figure S19. High-resolution scans of the O1s electrons of BTO-Q before and after N_2 reduction reaction.

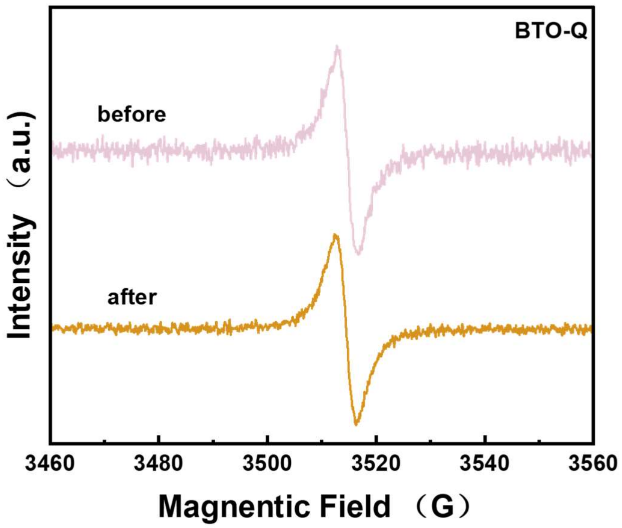

Figure S20. EPR spectra of BTO-Q before and after photocatalytic NRR.

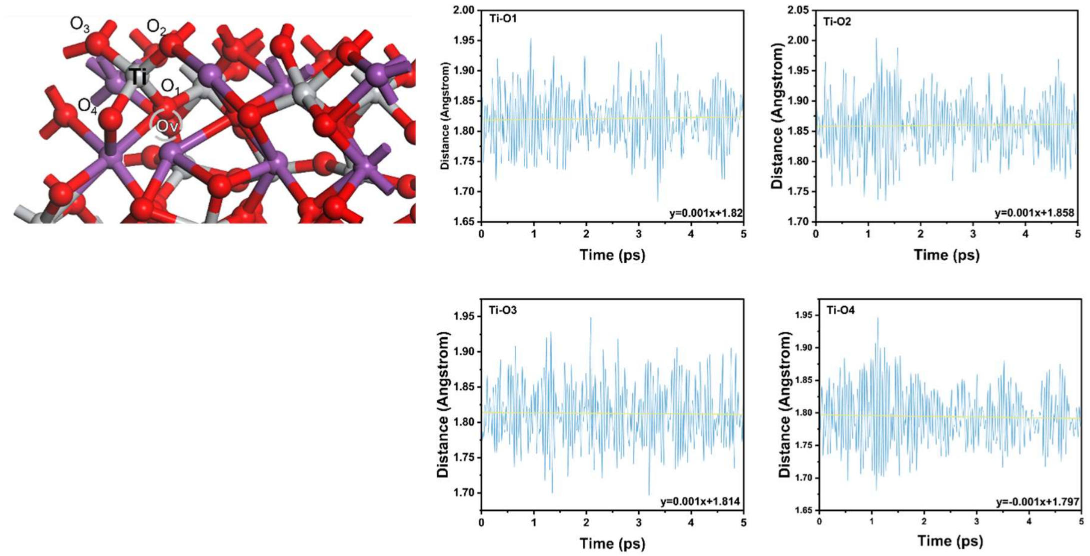

Figure S21. Distance between the Ti and O atoms versus the ab initio molecular dynamics time for BTO (110) with oxygen vacancies.

The original bond lengths of Ti-O1, Ti-O2, Ti-O3, and Ti-O4 are 1.82 Å, 1.85 Å, 1.79 Å, and 1.77 Å, respectively. The fitted curves indicate that the chemical bonds between titanium and the four oxygen atoms are centered around 1.82 Å, 1.86 Å, 1.81 Å, and 1.80 Å, respectively, on the time scale, and the variations in bond lengths are all within reasonable limits.

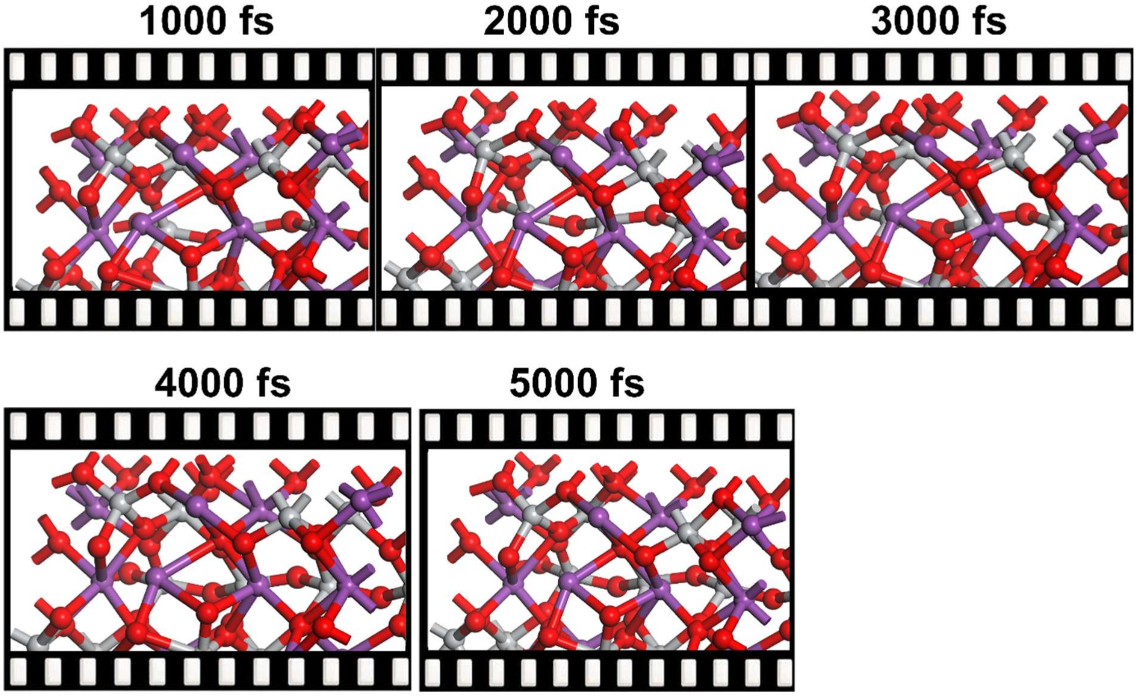

Figure S22. Snapshots of BTO (110) with oxygen vacancies in molecular dynamic simulation.

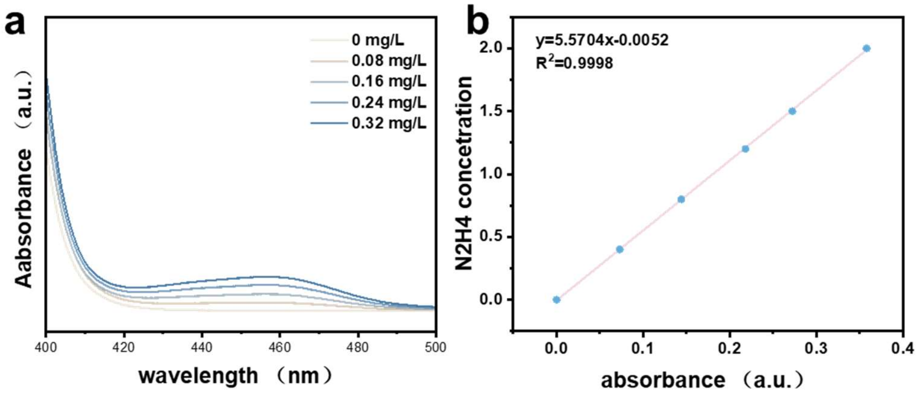

Figure S23. (a) Absorbance spectra of $N_2H_4 \cdot H_2SO_4$ standard solutions, and (b) the corresponding calibration curve for $N_2H_4 \cdot H_2SO_4$.

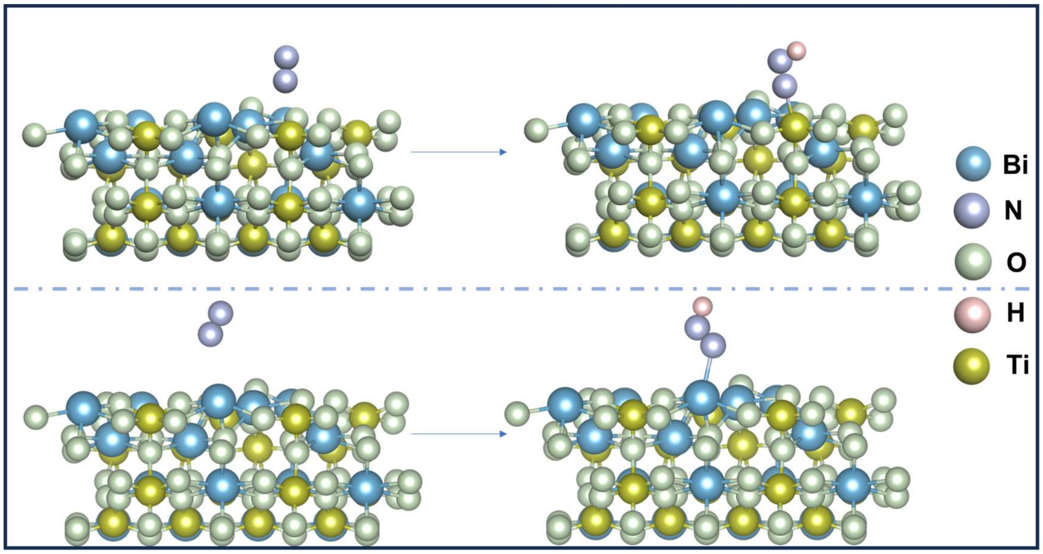

Figure S24. N_2 adsorption on $Bi_2Ti_2O_7$ (110) in oxygen-containing vacancies

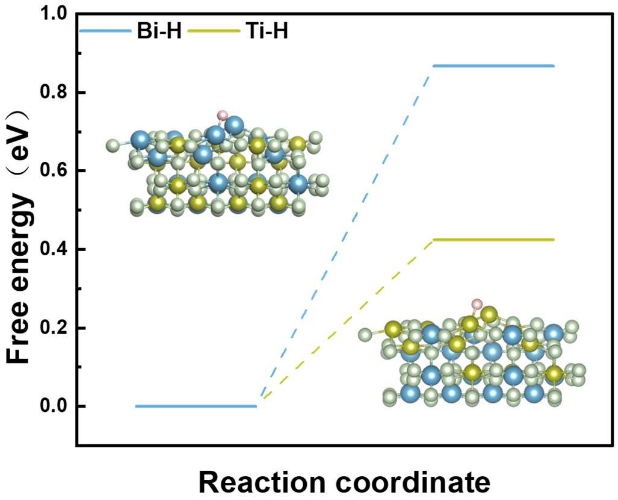

Figure S25. *H free energies and bond lengths of BTO (110) with oxygen vacancies.

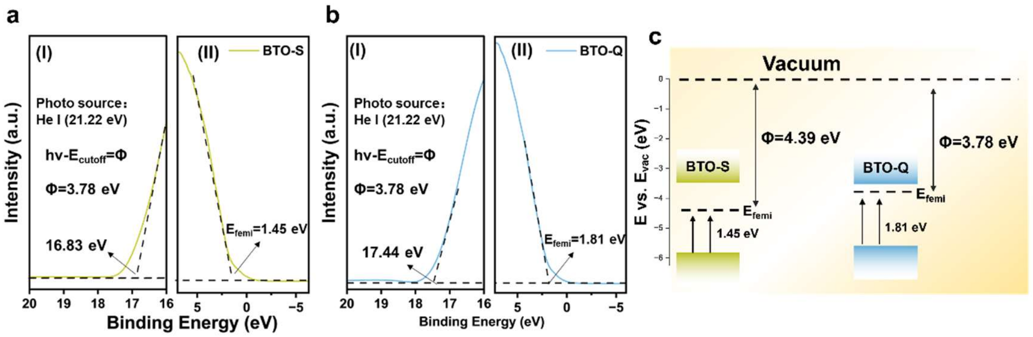

Figure S26. UPS spectra of (a) BTO-S and (b) BTO-Q at (I) -5 V bias and (II) no bias. (c) Energy band positions of BTO-Q and BTO-S relative to the vacuum energy level.

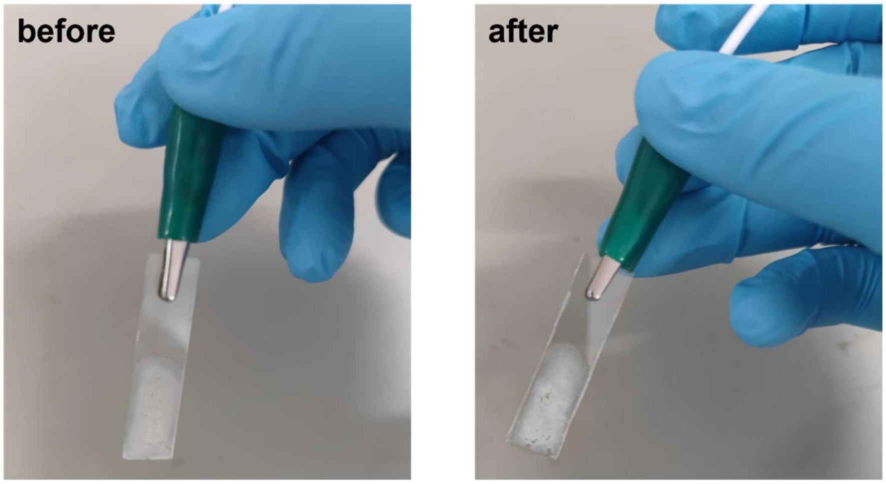

Figure S27. Photographs of the sample appearance before and after electrochemical testing.

Table S1. Fitting parameters of the Bi L_{III}-edge EXAFS of BTO-Q.

Path	R (Å)	N	ΔE_0 (eV)	R-factor
Bi-O-Ti	2.57	0.96 ± 0.28	-7.71 ± 2.73	0.029

Table S2. Fitting parameters of the Bi L_{III}-edge EXAFS of BTO-S.

Path	R (Å)	N	ΔE_0 (eV)	R-factor
Bi-O-Ti	2.69	0.98 ± 0.35	-9.07 ± 6.15	0.011

Table S3. NH_4Cl standard solutions and the absorbance at 420 nm by indophenol assay.

$C_{standard}$ (mg L^{-1})	V_A (mL)	V_{water} (mL)	Absorbance (420 nm)
0.0	0.0	5	0.023
0.4	1	4	0.096
0.8	2	3	0.157
1.2	3	2	0.241
1.5	3.75	1.25	0.305
2	5.0	0	0.378

Table S4. Ammonia concentration detected by ion chromatography

Sample	Ion	Residence time (min)	Peak area (μS*min)
BTO-Q	NH_4^+	4.57	1.2322
BTO-S	NH_4^+	4.57	0.1421

Table S5. Comparison of the N_2 fixation activity of BTO-Q with those of relevant literature results.

Catalyst	Scavenger	AQE (%)	NH_3 ($\mu mol\ g^{-1}h^{-1}$)	Ref.
$Bi/Bi_2Sn_2O_7$	No	-	284.5	[17]
$MoS_2/In\text{-}Bi_2MOO_6$	No	365 nm-1.2<br>380 nm-0.81<br>400 nm-0.61<br>420 nm-0.33<br>450 nm-0.12<br>500 nm-0.09	90	[18]
$BiOBr/Bi_4O_5Br_2$	No	365 nm-0.15<br>400 nm-0.05<br>420 nm-0.04<br>500 nm-0.02	66.87	[19]
ultrathin $Bi_4O_5Cl_2$ nanosheets	No	-	112.6	[20]
MIL-125(Ti)	No	-	156.9	[21]
$Co\text{-}Bi_2MoO_6$	No	365 nm-1.009<br>380 nm-0.806<br>400 nm-0.561<br>420 nm-0.224<br>450 nm-0.172<br>500 nm-0.074<br>550 nm-0.02	95.5	[22]
Bi4O5Br2/CdWO4	Methanol	-	501	[23]
Bi2MoO6/OV–BiOBr	No	-	90.7	[24]
Ultrathin Bi4O5Br2	No	-	109.0	[25]
Fe−BiOBr	No	365 nm-0.6<br>420 nm-0.16	46.1	[26]
ZnIn2S4/BiOCl	No	-	14.6	[27]
Fe−BiOCl	No	-	30	[28]
In2O3/In2S3	No	-	40.04	[29]
Ni2P+BP	methanol	420 nm-0.46	6.14	[30]
ZnCr-LDH	No	-	33.19	[31]
defect-rich Bi3O4Br	No	-	50.8	[32]
VO-BiOBr	No	-	49.04	[33]
MoO3−x	No	-	11.1	[34]
Au@UiO66/PTFE membrane	No	520 nm-1.54	380	[35]
BTO-Q	No	365 nm-1.13<br>420 nm-0.61<br>490 nm-0.36<br>550 nm-0.12	332.03	This work

Table S6. Fitted results of the TRPL data of BTO-Q and BTO-S with a biexponential equation.

	τ_1 (ns)	τ_2 (ns)	A_1 (%)	A_2 (%)	τ_{ave} (ns)
BTO-Q	1.55	8.53	114.02	27.49	5.53
BTO-S	1.09	4.13	67.85	16.68	2.56

References

[1] L. D. Geoffrion,G. Guisbiers. Journal of Physics and Chemistry of Solids, 2020, 140: 109320.

[2] P. Giannozzi, S. Baroni, N. Bonini, M. Calandra, R. Car, C. Cavazzoni, D. Ceresoli, G. L. Chiarotti, M. Cococcioni, I. Dabo, A. Dal Corso, S. de Gironcoli, S. Fabris, G. Fratesi, R. Gebauer, U. Gerstmann, C. Gougoussis, A. Kokalj, M. Lazzeri, L. Martin-Samos, N. Marzari, F. Mauri, R. Mazzarello, S. Paolini, A. Pasquarello, L. Paulatto, C. Sbraccia, S. Scandolo, G. Sclauzero, A. P. Seitsonen, A. Smogunov, P. Umari,R. M. Wentzcovitch. Journal of Physics: Condensed Matter, 2009, 21(39): 395502.

[3] P. Giannozzi, O. Andreussi, T. Brumme, O. Bunau, M. Buongiorno Nardelli, M. Calandra, R. Car, C. Cavazzoni, D. Ceresoli, M. Cococcioni, N. Colonna, I. Carnimeo, A. Dal Corso, S. de Gironcoli, P. Delugas, R. A. DiStasio, A. Ferretti, A. Floris, G. Fratesi, G. Fugallo, R. Gebauer, U. Gerstmann, F. Giustino, T. Gorni, J. Jia, M. Kawamura, H. Y. Ko, A. Kokalj, E. Küçükbenli, M. Lazzeri, M. Marsili, N. Marzari, F. Mauri, N. L. Nguyen, H. V. Nguyen, A. Otero-de-la-Roza, L. Paulatto, S. Poncé, D. Rocca, R. Sabatini, B. Santra, M. Schlipf, A. P. Seitsonen, A. Smogunov, I. Timrov, T. Thonhauser, P. Umari, N. Vast, X. Wu,S. Baroni. Journal of Physics: Condensed Matter, 2017, 29(46): 465901.

[4] J. P. Perdew, A. Ruzsinszky, G. I. Csonka, O. A. Vydrov, G. E. Scuseria, L. A. Constantin, X. Zhou,K. Burke. Physical Review Letters, 2008, 100(13): 136406.

[5] G. Prandini, A. Marrazzo, I. E. Castelli, N. Mounet,N. Marzari. npj Computational Materials, 2018, 4(1): 72.

[6] K. Lejaeghere, G. Bihlmayer, T. Björkman, P. Blaha, S. Blügel, V. Blum, D. Caliste, I. E. Castelli, S. J. Clark, A. Dal Corso, S. de Gironcoli, T. Deutsch, J. K. Dewhurst, I. Di Marco, C. Draxl, M. Dułak, O. Eriksson, J. A. Flores-Livas, K. F. Garrity, L. Genovese, P. Giannozzi, M. Giantomassi, S. Goedecker, X. Gonze, O. Grånäs, E. K. U. Gross, A. Gulans, F. Gygi, D. R. Hamann, P. J. Hasnip, N. A. W. Holzwarth, D. Iuşan, D. B. Jochym, F. Jollet, D. Jones, G. Kresse, K. Koepernik, E. Küçükbenli, Y. O. Kvashnin, I. L. M. Locht, S. Lubeck, M. Marsman, N. Marzari, U. Nitzsche, L. Nordström, T. Ozaki, L. Paulatto, C. J. Pickard, W. Poelmans, M. I. J. Probert, K. Refson, M. Richter, G.-M. Rignanese, S. Saha, M. Scheffler, M. Schlipf, K. Schwarz, S. Sharma, F. Tavazza, P. Thunström, A. Tkatchenko, M. Torrent, D. Vanderbilt, M. J. van Setten, V. Van Speybroeck, J. M. Wills, J. R. Yates, G.-X. Zhang,S. Cottenier. Science, 2016, 351(6280): aad3000.

[7] S. Grimme, J. Antony, S. Ehrlich,H. Krieg. The Journal of Chemical Physics, 2010, 132(15).

[8] N. Marzari, D. Vanderbilt, A. De Vita,M. C. Payne. Physical Review Letters, 1999, 82(16): 3296-3299.

[9] P. Li, C. Jiang, Q. Wang, K. Zuojiao, Z. Zhang, J. Liu,Y. Wang. Journal of Nanoscience and Nanotechnology, 2018, 18(12): 8360-8366.

[10] J. K. Nørskov, J. Rossmeisl, A. Logadottir, L. Lindqvist, J. R. Kitchin, T. Bligaard,H. Jónsson. The Journal of Physical Chemistry B, 2004, 108(46): 17886-17892.

[11] P. Liu, C. Fu, Y. Li,H. Wei. Physical Chemistry Chemical Physics, 2020, 22(17): 9322-9329.

[12] C. Turner, P. M. Johns, E. M. Thatcher, D. B. Tanner,J. C. Nino. The Journal of Physical Chemistry C, 2014, 118(49): 28797-28803.

[13] Y. Zhang, J. Di, X. Qian, M. Ji, Z. Tian, L. Ye, J. Zhao, S. Yin, H. Li,J. Xia. Applied Catalysis B: Environmental, 2021, 299: 120680.

[14] S. Z. Andersen, V. Čolić, S. Yang, J. A. Schwalbe, A. C. Nielander, J. M. McEnaney, K. Enemark-Rasmussen, J. G. Baker, A. R. Singh, B. A. Rohr, M. J. Statt, S. J. Blair, S. Mezzavilla, J. Kibsgaard, P. C. K. Vesborg, M. Cargnello, S. F. Bent, T. F. Jaramillo, I. E. L. Stephens, J. K. Nørskov,I. Chorkendorff. Nature, 2019, 570(7762): 504-508.

[15] L. Ma, Y. Cheng, G. Cavataio, R. W. McCabe, L. Fu,J. Li. Chemical Engineering Journal, 2013, 225: 323-330.

[16] Y. Li, Z. Wang, H. Ji, L. Zhang, T. Qian, C. Yan,J. Lu. Chinese Journal of Catalysis, 2023, 44: 50-66.
[17] R. Wu, S. Gao, C. Jones, M. Sun, M. Guo, R. Tai, S. Chen,Q. Wang. Advanced Functional Materials, n/a(n/a): 2314051.
[18] T. Ma, R. Li, Y.-C. Huang, Y. Lu, L. Guo, M. Niu, X. Huang, R. A. Soomro, J. Ren, Q. Wang, B. Xu, C. Yang, F. Fu,D. Wang. ACS Catalysis, 2024, 14(8): 6292-6304.
[19] H. Wang, Z. Chen, Y. Shang, C. Lv, X. Zhang, F. Li, Q. Huang, X. Liu, W. Liu, L. Zhao, L. Ye, H. Xie,X. Jin. ACS Catalysis, 2024, 14(8): 5779-5787.
[20] X. a. Dong, X. Shi, Z. Cui, W. Dai,F. Dong. ACS Nano, 2024, 18(13): 9670-9677.
[21] Y. Sun, H. Ji, Y. Sun, G. Zhang, H. Zhou, S. Cao, S. Liu, L. Zhang, W. Li, X. Zhu,H. Pang. Angewandte Chemie International Edition, 2024, 63(3): e202316973.
[22] Y. Chunming, Z. Yuanyuan, Y. Feng, D. Rui, M. Taoxia, B. Yujie, L. Ruqi, G. Li, W. Danjun,F. Feng. Applied Catalysis B: Environmental, 2023.
[23] C. Zhao, X. Li, L. Yue, X. Ren, S. Yuan, Z. Zeng, X. Hu, Y. Wu,Y. He. ACS Applied Nano Materials, 2023, 6(17): 15709-15720.
[24] X. Xue, R. Chen, C. Yan, Y. Hu, W. Zhang, S. Yang, L. Ma, G. Zhu,Z. Jin. Nanoscale, 2019, 11(21): 10439-10445.
[25] X. a. Dong, Z. Cui, X. Shi, P. Yan, Z. Wang, A. C. Co,F. Dong. Angewandte Chemie International Edition, 2022, 61(19): e202200937.
[26] X. Chen, X. Zhang, Y.-H. Li, M.-Y. Qi, J.-Y. Li, Z.-R. Tang, Z. Zhou,Y.-J. Xu. Applied Catalysis B: Environmental, 2021, 281: 119516.
[27] R. Jiang, D. Wu, G. Lu, Z. Yan,J. Liu. Chemosphere, 2019, 227: 82-92.
[28] Z. Shen, F. Li, J. Lu, Z. Wang, R. Li, X. Zhang, C. Zhang, Y. Wang, Y. Wang, Z. Lv, J. Liu,C. Fan. Journal of Colloid and Interface Science, 2021, 584: 174-181.
[29] H. Xu, Y. Wang, X. Dong, N. Zheng, H. Ma,X. Zhang. Applied Catalysis B: Environmental, 2019, 257: 117932.
[30] Z.-K. Shen, M. Cheng, Y.-J. Yuan, L. Pei, J. Zhong, J. Guan, X. Li, Z.-J. Li, L. Bao, X. Zhang, Z.-T. Yu,Z. Zou. Applied Catalysis B: Environmental, 2021, 295: 120274.
[31] Y. Zhao, Y. Zhao, G. I. N. Waterhouse, L. Zheng, X. Cao, F. Teng, L.-Z. Wu, C.-H. Tung, D. O'Hare,T. Zhang. Advanced Materials, 2017, 29(42): 1703828.
[32] J. Di, J. Xia, M. F. Chisholm, J. Zhong, C. Chen, X. Cao, F. Dong, Z. Chi, H. Chen, Y.-X. Weng, J. Xiong, S.-Z. Yang, H. Li, Z. Liu,S. Dai. Advanced Materials, 2019, 31(28): 1807576.
[33] X. Xue, R. Chen, H. Chen, Y. Hu, Q. Ding, Z. Liu, L. Ma, G. Zhu, W. Zhang, Q. Yu, J. Liu, J. Ma,Z. Jin. Nano Letters, 2018, 18(11): 7372-7377.
[34] Y. Li, X. Chen, M. Zhang, Y. Zhu, W. Ren, Z. Mei, M. Gu,F. Pan. Catalysis Science & Technology, 2019, 9(3): 803-810.
[35] L.-W. Chen, Y.-C. Hao, Y. Guo, Q. Zhang, J. Li, W.-Y. Gao, L. Ren, X. Su, L. Hu, N. Zhang, S. Li, X. Feng, L. Gu, Y.-W. Zhang, A.-X. Yin,B. Wang. Journal of the American Chemical Society, 2021, 143(15): 5727-5736.
